# From Diabetes to Degenerative Diseases: The Multifaceted Action of Metformin

**DOI:** 10.3390/ijms26199748

**Published:** 2025-10-07

**Authors:** Lucrezia Irene Maria Campagnoli, Angelica Varesi, Foroogh Fahmideh, Reza Hakimizad, Petra Petkovic, Annalisa Barbieri, Nicoletta Marchesi, Alessia Pascale

**Affiliations:** 1 Department of Drug Sciences, Section of Pharmacology, University of Pavia, 27100 Pavia, Italy; lucreziairenem.campagnoli01@universitadipavia.it (L.I.M.C.); foroogh.ft@gmail.com (F.F.); petra.petkovic01@universitadipavia.it (P.P.); annalisa.barbieri@unipv.it (A.B.); alessia.pascale@unipv.it (A.P.); 2 Department of Molecular Genetics, University of Toronto, Toronto, ON M5G 1L7, Canada; angelica.varesi@mail.utoronto.ca; 3 Princess Margaret Cancer Centre, Toronto, ON M5G 2M9, Canada; 4 School of Medicine and Surgery, University of Milano Bicocca, 20126 Milan, Italy; hakimizad.r@gmail.com

**Keywords:** metformin, diabetes mellitus, age-related disorders, Alzheimer’s disease, Parkinson’s disease, osteoporosis

## Abstract

Metformin, an oral antihyperglycemic drug, represents the cornerstone of pharmacological treatment for type 2 diabetes mellitus (T2DM). Its primary glucose-lowering effects are well established, predominantly mediated through the activation of AMP-activated protein kinase (AMPK). This activation leads to a reduction in hepatic glucose production (primarily by inhibiting gluconeogenesis and glycogenolysis) and an increase in peripheral glucose uptake and utilization. Beyond its direct impact on glucose metabolism, metformin also improves insulin sensitivity and has beneficial effects on lipid profiles. Increasingly, research shows that metformin has pleiotropic effects. In addition to its recognized antihyperglycemic action, metformin is emerging as a regulator of cellular processes implicated in aging. Indeed, emerging evidence suggests a potential role of metformin in modulating pathways associated with longevity and ameliorating the symptoms of age-related diseases, including neurodegenerative disorders (such as Alzheimer’s and Parkinson’s diseases), cardiovascular diseases, age-related macular degeneration, and osteoporosis. The proposed mechanisms for these broader effects involve AMPK activation, modulation of the mTOR pathway, reduction of oxidative stress, and promotion of autophagy. After exploring the established role of metformin in T2D, this review provides a comprehensive investigation of its promising applications in the context of age-related diseases, offering valuable insights into its multifaceted therapeutic potential beyond glycemic control.

## 1. Introduction

Metformin (*N*,*N*-dimethyl biguanide), a biguanide derivative, is an oral hypoglycaemic drug originally derived from the traditional herbal medicine *Galega officinalis*, is rich in guanidine, and is known to lower blood glucose. In 1957, the French physician Jean Sterne introduced this drug as an antihyperglycemic agent. Since then, it has become one of the most widely used pharmaceutical interventions worldwide due to its clinical safety, high tolerability, robust glucose reduction, low cost, and broad availability [1,2,3]. Following oral administration, metformin is primarily absorbed in the small intestine, with a bioavailability of approximately 50–60%. Several organic cationic transporters, such as organic cation transporters novel 1 (OCTN1), OCTN2, and plasma membrane monoamine transporter (PMAT), mediate its uptake and excretion. The drug is primarily excreted in the urine, with a half-life of roughly five hours, while the remaining portion enters the colon and is excreted in the faeces. The most common side effects include gastrointestinal disturbances (diarrhea, nausea, and/or abdominal discomfort), folate and vitamin B12 deficiency, and lactic acidosis [2]. Overall, metformin has low interaction with other drugs. However, some drugs such as cimetidine, topiramate, ranolazine, and cephalexin, compete with metformin during renal excretion and increase the risk of developing lactic acidosis. Other drugs, such as aspirin and antidiabetic agents, increase the risk of hypoglycemia when used with metformin [4]. According to international guidelines published by the American Diabetes Association (ADA) and the European Association for the Study of Diabetes (EASD), metformin is recommended as the first-line drug for the treatment of individuals with type 2 diabetes mellitus (T2DM) at all stages of the disease [5]. Various preclinical and clinical studies demonstrated the ability of metformin to improve insulin sensitivity through several mechanisms, including activation of the insulin signaling pathway, activation of adenosine monophosphate (AMP)-activated protein kinase (AMPK; a key regulator of numerous metabolic pathways, including glucose metabolism, lipid metabolism, and energy homeostasis), epigenetic modifications, and improvements in the translocation of glucose transporter 4 (GLUT-4) to the plasma membrane. Mechanistically, metformin inhibits mitochondrial complex I, leading to adenosine triphosphate (ATP) depletion and a rise in AMP intracellular levels. This results in the activation of AMPK, which in turn alters the cellular redox potential [6,7]. A similar mechanism can be activated independently of AMP. Indeed, a recent study demonstrated that low concentrations of metformin (5 μM) activate AMPK through inhibition of the ATPase H+ transporting accessory protein 1 (ATP6AP1) of the lysosomal V-ATPase complex [8]. The choice of metformin’s mechanism of action is dose-dependent: high concentrations inhibit the respiratory chain complex I, leading to an increase in the AMP/ATP ratio, whereas low doses are insufficient to increase this ratio [9,10]. Initially, it was believed that metformin primarily affected the hepatic mitochondrial complex I; however, subsequent research has proposed additional mitochondrial targets, such as mitochondrial glycerol-3-phosphate dehydrogenase (mGPDH) and complex IV. Nevertheless, the significance of these alternative mechanisms remains controversial [11].

Beyond its established role in T2DM management, the pleiotropic actions of metformin have been extensively documented. Indeed, metformin is a widely used clinical drug with numerous benefits, including antiviral [12], anti-inflammatory [13], and antioxidant [14] properties. Its potential use has also been documented in cancer [15]; liver [16], neurological [17], and renal [18] diseases; polycystic ovary syndrome [19]; and obesity [20]. Furthermore, metformin can influence the function of many cell types, including pancreatic β-cells [21,22,23]. Specifically, it has been shown to protect human β-cells by mitigating the stress induced by pro-inflammatory cytokines [24].

Given the increasing global population of elderly individuals, the development of effective therapeutic strategies to prevent and/or treat age-related diseases is of paramount importance. In this context, numerous studies have demonstrated the anti-aging effects of metformin by targeting key molecules associated with this irreversible biological process characterized by a progressive loss of physiological function, which leads to the onset of several age-related diseases, including neurodegenerative, cardiovascular, and skeletal disorders [25]. Compelling evidence indicates that metformin impacts aging hallmarks, such as mitochondrial dysfunction, cellular senescence, telomere attrition, genomic instability, loss of proteostasis, and dysregulated nutrient sensing [26]. For instance, this drug has been reported to activate the sirtuin 3 (SIRT3)-AMPK pathway and increase peroxisome proliferator-activated receptor-gamma coactivator 1 alpha (PGC-1α) expression, thereby promoting mitochondrial biogenesis. It also inhibits the senescence-associated secretory phenotype (SASP) and cellular senescence in multiple age-related dysfunctions. The drug prevents telomere shortening and genomic instability by partly inhibiting oxidative stress (OS) and DNA damage and regulating ataxic telangiectasis mutation (ATM) protein kinase. Furthermore, it maintains proteostasis through the inhibition of protein misfolding and enhancement of autophagy. Finally, it improves dysregulated nutrient sensing primarily through the down-regulation of the insulin/insulin-like growth factor 1 (IGF-1) and mammalian target of rapamycin complex I (mTORC1), as well as the up-regulation of AMPK and SIRT1 nutrient-sensing signaling pathways [25,26,27,28,29,30]. Notably, the 6-year double-blind, placebo-controlled, multicenter clinical trial Targeting Aging with Metformin (TAME) has recently been proposed to investigate metformin’s anti-aging effect. Specifically, this study was designed to include approximately 3000 men and women aged 65–80 years, without diabetes while at high risk of age-related diseases, to determine if treatment with metformin (1500 mg/day) can prevent the onset of multiple age-related diseases and other aging phenotypes [31].

Building upon this evidence, this manuscript first focuses on the main mechanisms of metformin in treating T2DM and subsequently aims to investigate its potential therapeutic effects in preventing or managing age-related disorders, including neurodegenerative disorders, such as Alzheimer’s disease and Parkinson’s disease, cardiovascular diseases, osteoporosis, and age-related macular degeneration.

## 2. Metformin in T2DM: A Longstanding Therapeutic Application

Numerous studies and clinical trials have widely demonstrated the use of metformin in T2DM treatment. Metformin is used either as monotherapy or in combination with other hypoglycemic agents such as sulfonylureas, dipeptidyl peptidase-4 inhibitors, sodium-glucose transport protein-2 inhibitors, and insulin, which exerts synergistic effects [5,32,33,34,35,36,37,38]. Furthermore, this drug has been proposed as preventive therapy in pre-diabetic individuals as well as a potential treatment for patients with type 1 diabetes (T1DM) [39,40]. Its preventative action has recently been described [39]. Moreover, it has been reported that some patients with T1DM, despite insulin therapy, are unable to achieve adequate glycemic control. Those patients exhibit significantly high levels of HbA1c (glycated hemoglobin; an indicator of average blood sugar levels over the past 2–3 months) therefore necessitating higher insulin dosages. Given that metformin is known to counteract insulin resistance, its adjunctive administration could be a viable therapeutic option [40].

**Figure 1:**
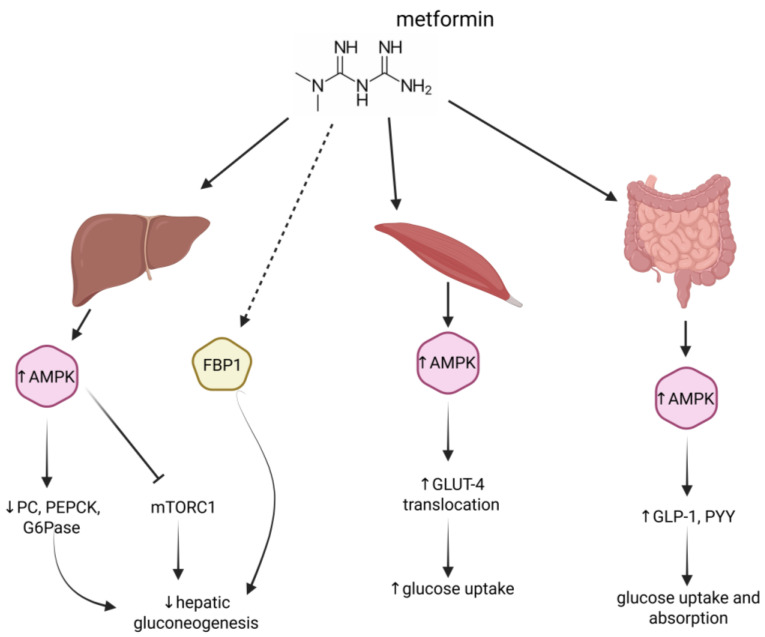
Mechanisms of action of metformin in T2DM. Metformin exerts its antihyperglycemic effects at three different levels: at the hepatocyte level by reducing hepatic gluconeogenesis; at the skeletal muscle level by increasing GLUT-4-mediated glucose uptake; at the gastrointestinal tract by affecting glucose uptake and absorption. Abbreviations: AMPK: 5′ AMP-activated protein kinase; FBP1: fructose-1,6-bisphosphatase-1; G6Pase: glucose 6 phosphatase; GLP-1: glucagon-like peptide-1; GLUT-4: glucose transporter 4; mTORC1: mammalian target of rapamycin complex 1; PC: pyruvate carboxylase; PEPCK: phosphoenolpyruvate carboxykinase; PYY: peptide YY. (Created with Biorender 2025).

Although metformin’s mechanisms of action are known, they still remain in part unclear and an ongoing subject of research [2]. Overall, this biguanide drug acts as described below (Figure 1).

**At the hepatocyte level**, metformin acts by reducing hepatic gluconeogenesis and glycogenolysis through AMPK-dependent [41,42] or AMPK-independent pathways [43,44]. One of the main antidiabetic mechanisms identified is the inhibition of mitochondrial respiratory chain complex I [45]. The evidence that complex I is one of the primary target of metformin has recently been strengthened by the demonstration that the metabolic effect of the drug is preserved in mice deficient in liver-specific AMPK [11]. Specifically, after oral administration, metformin enters liver cells via the OCT1 (organic cation transporter 1) transporter, highly expressed on the membrane of hepatocytes in the periportal area of the liver [46]. This particular specificity of the channel’s localization also explains the drug’s highly selective effects. Being a positively charged molecule, it accumulates primarily in the mitochondria, where it blocks complex I and activates the protein kinase AMPK.

Specifically, metformin suppresses the expression of gluconeogenic genes, such as phosphoenolpyruvate carboxykinase, glucose 6 phosphatase, and pyruvate carboxylase by inhibiting gluconeogenesis through AMPK-dependent activation of small heterodimer partner (a member of the nuclear receptor superfamily) and inhibition of cAMP response element-binding protein (CREB) phosphorylation [47,48]. Furthermore, it may also suppress hepatic gluconeogenesis through AMPK-dependent inhibition of the protein kinase mTORC1 [49]. Metformin can also decrease hepatic glucose production in an AMPK-independent manner. In this regard, a recent study reported that metformin achieves this by directly bind fructose-1,6-bisphosphatase-1 enzyme, leading to a reduction in hepatic glucose production [50]. Additionally, other studies have revealed its effect in attenuating glucagon activity [51], inhibiting mGPDH [44], and lowering plasma glucose levels by enhancing GLUT-1-mediated glucose transport into hepatocytes through insulin receptor substrate 2 (IRS-2) activation [52]. Overall, these effects lead to a reduction in hepatic sugar production, improved glucose utilization in muscles, a decrease in fat synthesis (lipogenesis), and an increase in fat breakdown (lipolysis).

**At the skeletal muscle level**, metformin acts by increasing GLUT-4-mediated glucose uptake, thereby lowering plasma glucose levels. In this context, a recent study highlights that metformin appears to increase peripheral glucose utilization by inducing GLUT-4 expression and enhancing its translocation to the plasma membrane, also through changes in insulin signaling pathway mediators, AMPK activation, and epigenetic variations. Moreover, numerous animal and human studies have indicated that impaired GLUT-4-mediated glucose uptake is a primary mechanism underlying insulin resistance [53]. A molecular analysis of the mechanism of GLUT-4 action reveals an important distinction between the transporter’s translocation and its subsequent functional activation. GLUT-4 translocation to the plasma membrane alone is not sufficient to initiate glucose transport. The use of a phosphoinositide-binding peptide (PBP10) induced the translocation of GLUT-4 in an inactive form [54], demonstrating that additional insulin stimulation is required for glucose transport activation. Both processes, however, appear to depend on PI3K activity. This duality in the GLUT-4 regulatory mechanism suggests potential complexity in how drugs like metformin can influence glucose uptake by acting on one or both aspects of this crucial process.

**In the gastrointestinal tract**, metformin acts by affecting glucose uptake and absorption, promoting the transport of lactate to the liver, increasing glucagon-like peptide-1 (GLP-1) and peptide YY (PYY) secretion through the intestinal AMPK-dependent pathway, and modifying the gut microbiota (GM) composition [55]. As this regard, it has also been shown that the increase in GLP-1 is due to an inhibition of the DPP4 enzyme, which also regulates the levels of peptide YY. In particular, it has been observed that the DPP4 enzyme, produced by the intestinal microbiota, is capable of metabolizing human GLP-1, which is potentially active in cases of impaired intestinal permeability due to inflammation, a characteristic of diabetes [55].

Notably, an increasing number of studies have demonstrated GM dysbiosis in patients with T2DM and reported metformin’s effect in influencing GM composition and function, providing new insights into the mechanisms underlying the anti-diabetic effects of metformin [56,57,58,59]. Specifically, the drug has been observed to increase the abundance of beneficial bacteria, such as *Akkermansia muciniphila*, known for their positive effects on gut health, influence gene expression of certain bacterial species, helping to maintain GM balance, and stimulate the production of short-chain fatty acids (SCFAs), which are important for intestinal health and metabolic regulation [59]. Furthermore, another study reported a reduction in *Bacteroides fragilis*, which resulted in improved glucose tolerance [60]. However, metformin’s potential impact on GM has been primarily studied in preclinical models, and its effects on human GM remain uncertain. Nevertheless, the addition of probiotics to metformin therapy has been associated with improved T2DM outcomes [61].

## 3. Multifaceted Metformin: Exploring Its Role in Age-Related Diseases

### 3.1. Alzheimer’s Disease

**Figure 2:**
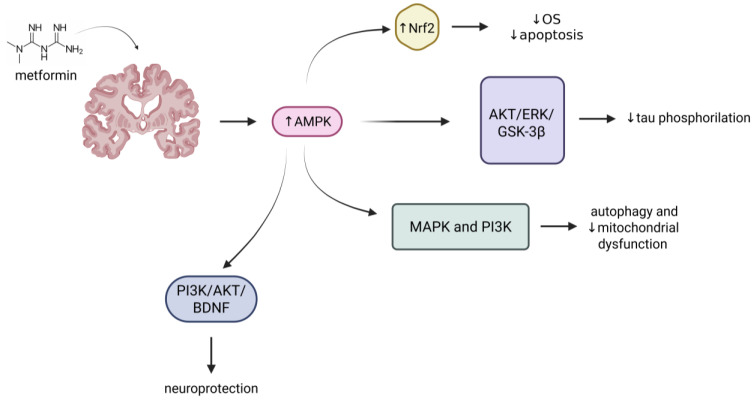
Potential mechanisms of action of metformin in AD. Abbreviations: AMPK: 5′ AMP-activated protein kinase; AKT: protein kinase B; BDNF: brain-derived neurotrophic factor; ERK: extracellular signal-regulated kinase; GSK-3β: glycogen synthase kinase-3β; MAPK: mitogen-activated protein kinase; Nrf2: nuclear factor erythroid 2-related factor 2; OS: oxidative stress; PI3K: phosphatidylinositol 3-kinase (Created with Biorender).

Alzheimer’s disease (AD) is the most prevalent form of neurodegeneration, and its incidence is projected to rise with population aging. While numerous therapeutic strategies have been proposed and tested, effective and curative interventions for AD remain elusive. Recent epidemiology studies have revealed a significant correlation between the incidence of AD and T2DM (Table 1) [62,63,64,65,66,67,68,69,70,71,72,73,74,75,76,77], providing a rationale for investigating metformin as a potential treatment for dementia [78]. Being commonly prescribed as a hypoglycemic drug, metformin is safe and well-tolerated, with no significant side effects reported in individuals with Mild Cognitive Impairment (MCI) or AD [72,73]. Thanks to its ability to efficiently cross the blood–brain barrier (BBB), metformin rapidly accumulates in the brain, where it exerts anti-inflammatory and antioxidant effects [79,80]. In this regard, it is crucial to note that, in AD patients, the typical elevated cholesterol levels, coupled with abnormal sphingolipid and phospholipid production, damage key cells in the brain, including pericytes, vascular endothelial cells, microglia, and astrocytes [81,82]. Thus, the neuroprotective effects of metformin might be significantly beneficial in mitigating these damages. This is particularly relevant in diabetic patients, where hyperglycemia triggers OS, with consequent accumulation of amyloid beta (Aβ) oligomers, neuronal toxicity, and brain inflammation [79,80]. Molecularly (Figure 2), metformin acts as a modulator of the AKT/ERK/GSK-3β signaling pathway and leverages GSK-3β suppression to reduce tau phosphorylation [83]. Though Nrf2 stabilization, metformin also mitigates oxidative stress and reduces cellular apoptosis [84]. Furthermore, by regulating the mitogen-activated protein kinase (MAPK) and phosphoinositide 3-kinase (PI3K) signaling cascades, metformin stimulates autophagy and counteracts mitochondrial dysfunction in the brain, thereby reducing AD risk [85,86]. The parallel modulation of IGF-1 and mTOR pathways further explains the positive effect of metformin intake in reducing AD occurrence, positioning this caloric restriction mimetic a strong candidate for clinical investigation [87,88]. Deeper insights gained from mouse studies have significantly advanced the mechanistic understanding of metformin in brain diseases. Notably, single-cell RNA sequencing in drug-treated amyloid precursor protein/presenilin 1 (APP/PS1) transgenic mice showed that negative regulation of apoptosis, enhanced astrocyte differentiation from stem cells, together with increased expression of neural stem cell and oligodendrocyte precursor cell self-renewal pathways resulted in improved spatial memory, reduced anxiety scores, and enhanced cognition [89]. Consistently, functional analysis demonstrated decreased hippocampal CA1 neuronal loss and reduced levels of the pro-apoptotic proteins caspase-3 and poly ADP ribose polymerase in animal models of AD treated with metformin [90,91,92,93]. In parallel, metformin improved cholinergic function by counteracting the reduced hippocampal acetylcholinesterase (AChE) activity typical of AD, thereby maintaining the neurotransmitter levels required to sustain memory and learning [90,93,94]. This was accompanied by reduced levels of Aβ oligomers and phosphorylated (p)-tau in the *corpus callosum* and hippocampus of treated animals compared to their untreated counterparts, which instead exhibited higher dementia scores [91,95,96]. Mechanistically, enhanced chaperone-mediated autophagy and phagocytosis in microglia are considered putative mechanisms contributing to Aβ plaque and tau protein clearance upon metformin administration [97,98]. Additionally, diminished neuroinflammation, through lowering levels of the pro-inflammatory cytokines tumor necrosis factor alpha (TNF-α) and interleukin 6 (IL-6), in the hippocampus of AD mice and rats under metformin therapy significantly reduced microgliosis and astrogliosis, with minimal glial fibrillary acidic protein (GFAP) and ionized calcium-binding adapter molecule 1 (IBA1) immunoreactivity [90,91,92,93,99,100]. Given the established correlation between inflammation and OS, it is noteworthy that while AD animals exhibited elevated OS parameters, as evidenced by increased levels of hippocampal inducible nitric oxide synthase (iNOS), neuronal nitric oxide synthase (nNOS), reactive oxygen species (ROS), malondialdehyde (MDA), and diminished superoxide dismutase (SOD), metformin reversed these effects [90,91,93,95,101]. Finally, and more intuitively, metformin mitigated insulin resistance by stimulating AMPK signaling, p-protein kinase B (p-AKT), protein kinase C (PKC), and p-glycogen synthase kinase (p-GSK)—all pathways and proteins whose activation promotes insulin sensitivity [91,95,96]. However, the role of metformin’s glucose-lowering effect in its therapeutic efficacy for AD remains debated, and further investigations are needed to understand whether blood sugar control is necessary or dispensable for its benefits in this context [96,99]. Nevertheless, animal studies across a variety of mouse and rat AD models consistently present compelling evidence supporting the use of metformin in clinical trials.

In humans, despite multiple meta-analyses and systematic reviews, including recent observational studies, reporting a lower risk of dementia associated with metformin intake, conflicting studies also exist [102,103,104]. For instance, while some studies, such as one involving 1873 T2DM patients with AD, showed a negative correlation between metformin use and the rate of cognitive decline [measured by mini-mental state examination (MMSE) score] [67], other research indicates that metformin intake may be even detrimental in aged AD patients, where alternative anti-diabetic drugs appear more effective in dementia prevention [69,70,74]. Critically, dose, duration, timings, and treatment formulations are crucial points to be considered when assessing the benefits of metformin across studies. This is further complicated by the fact that AD clinical trials primarily included T2DM patients, thus leaving the assessment of metformin intake in non-diabetic and healthy aged subjects unclear [105]. Furthermore, studies in mice subgroups have yielded conflicting results; indeed, while metformin improved attention, learning, and memory in healthy young mice, these effects were reversed upon chronic treatment in aged animals. Adding to these discrepancies, negative effects were observed in 3xTg-AD mice, where metformin correlated with higher levels of p-tau, Aβ plaques, and GSK-3β [106]. Similarly, enhanced p-tau, lipogenesis, and gliosis were associated with metformin administration in apolipoprotein E (APOE)-deficient mice [107]. Within this APOE genetic background, sex differences also appear to play a role, as metformin was reported to improve cognitive scores and behaviour in old female mice but proved detrimental in males [108]. In humans, studies indicate improved cognition in normal individuals taking metformin, but not in AD patients, with APOE genotype possibly acting as a confounder [68]. Beyond its potential as a curative treatment, the use of metformin in dementia prevention also remains debated [102,103,104]. For instance, no reduction in dementia occurrence was found in older diabetic patients taking metformin as a preventive strategy [77]. Nevertheless, this is likely due to the use of non-standardized definitions for risk/protection scores when evaluating therapeutic outcomes, which vary across therapeutic centres [63]. Moreover, the study’s limitation to Canadian participants introduces demographic, genetic background, environmental, and lifestyle biases, all recognized confounding factors. In this respect, a meta-analysis conducted on 100 reviews and 27 clinical studies reported a significant bias favouring metformin in Western countries, particularly the US, while no effect was observed in the Eastern populations [109]. Similar findings were reported in an independent meta-analysis, where no association between dementia risk and metformin intake was found among Europeans [110]. Conversely, metformin significantly decreased the risk of AD occurrence among US veterans under insulin therapy, as shown in an independent retrospective longitudinal study of 5528 participants [71]. In stark contrast, however, data from an observational study conducted on the Korean National Health Insurance Service DM cohort documented an increased risk of AD in T2DM patients undergoing metformin therapy, which was even worse among depressed participants [111]. Not only antidepressants but also non-steroidal anti-inflammatory drugs and neuropsychiatric conditions appear to alter the ability of metformin to reduce dementia risk, as reported by a longitudinal study involving 50 T2DM patients [76]. Lastly, it is important to consider that, as metabolism is shaped by various genetic, lifestyle, and environmental factors, geographical location might impact key molecular and functional targets of metformin, which can serve as a potential biomarker for sample stratification [112,113,114]. For instance, while cerebrospinal fluid (CSF) p-tau, total (t)-tau and Aβ-42 are potential biomarkers for metformin’s effect in diabetic patients [62], plasma and CSF levels of 7 proteins regulating inflammation and cell death (namely, REG1A, AZU1, PRTN3, CCL11, CCL20, CASP-2 and IL32, see Table 1) can monitor AD progression in non-diabetic MCI individuals [64]. Conversely, NDUFA2, a gene encoding for the nicotinamide adenine dinucleotide (NADH): ubiquinone oxidoreductase of mitochondrial complex I and a metformin target, was negatively correlated with AD risk in a Mendelian randomized cohort of over 500,000 participants [65].

Time-related assumptions made in observational studies, such as immortal time bias, time-window, or lag times, can further distort the result of clinical studies, leading to either an overestimation or underestimation of treatment effects [115]. In addition to these methodological challenges, the optimal doses and delivery methods are unclear and require standardization. Indeed, although oral administration is prevalent, intranasal delivery offers a potential advantage by increasing metformin concentration in the hippocampus compared to systemic circulation, which might be beneficial in AD patients without T2DM [116]. Even better, innovative formulations with improved BBB penetration, selective uptake by damaged neurons, and enhanced bioavailability, are now available and hold promise for future therapy optimization. These include, but are not limited to, supramolecular nanodrugs capable to release on-demand donepezil and metformin upon Aβ overexpression [117], mixed solid–liquid formulations [83], nanocarriers [118], and nanoflower multifunctional selenium carriers [119]. On this line, synergistic therapies combining metformin with reported natural compounds hold promise for further boosting therapeutic efficacy. For example, co-administration of the plant-derived cyanidin 3-O-galactoside with metformin showed synergistic activity in senescence-accelerated mouse-prone 8 (SAMP8) mice, reducing ketones, indoles, and Aβ-aggregation while increasing SCFA and fatty acid biosynthesis, thereby restoring memory and spatial learning capacity [120]. Similarly, reduced endoplasmic reticulum (ER) stress, achieved via protein kinase RNA-like ER kinase modulation, along with improved mitochondrial and metabolic functions was observed in SAMP8 mice through co-delivery of the alkaloid *Dendrobium nobile* Lindl. and metformin, respectively. In this study, reduced ER dilation coefficients and decreased calpain1, GSK-3β, and cyclin-dependent kinase 5 activity were identified as key markers of therapeutic efficacy [121]. Again, low doses of lupeol and metformin proved more effective than high-dose monotherapies, resulting in higher levels of the antioxidants nuclear factor erythroid 2-related factor 2 (Nrf2) and glutathione (GSH), along with lower myeloperoxidase, neutrophil neuroinflammation, OS, and AChE activity, ultimately improving Morris water maze scores in streptozotocin-induced mouse models of dementia [122].

A cross-sectional study in 50 individuals found that dipeptidyl peptidase 4 inhibition increased serum brain-derived neurotrophic factor (BDNF) and improved cognitive functions (as measured by standardized MMSE scores) in T2DM patients treated with metformin, calling for larger studies [123]. The ongoing MET-FINGER randomized double-blind phase IIb clinical trial, exploring the combination of multimodal lifestyle interventions (FINGER 2.0) with metformin in 600 participants aged between 60 and 79, promises to yield valuable insights. MET-FINGER will assess changes in memory, spatial learning, cognitive processing speed, metabolic and vascular status, and lifestyle wellbeing over 2 years, with a planned phase 3 clinical trial based on the results [124]. This trial is expected to confirm or disprove preliminary findings in diabetic AD rats, where aerobic exercise coupled with metformin was shown to improve cognitive function and hippocampal neurogenesis by reducing microgliosis and astrogliosis [125]. Therefore, although human studies are scarce, synergistic multimodal targeting appears as a promising approach for further enhancing metformin’s clinical benefits in dementia. In addition, innovative machine learning software applying artificial intelligence is emerging as a valuable tool to improve unbiased AD evaluation. As proof, a study conducted in 15,428 Swedish AD patients using unsupervised clustering to monitor cognitive performance demonstrated a significant correlation between metformin intake and cognitive function [75]. Given their unbiased evaluations, these tools are expected to play a crucial role in patient stratification and clinical monitoring, with the aim of improving standardization and supporting clinical decisions.

Despite emergent positive results, many cross-section studies and meta-analysis did not find any advantage in using metformin against AD. Indeed, while safe and tolerated, metformin was not associated with any improvement of AD or protection against dementia risk when adjusted for biases [72,73,103,115,126,127]. Moreover, the preferential inclusion for obese and diabetic participants further adds confounding factors, which makes the outcome difficult to interpret.

Overall, these data propose metformin as a safe, well-tolerated, commercially available, and effective therapy for reducing cognitive decline, sustaining memory and learning, and boosting attention. However, while mouse studies are generally positive, human clinical trials remain inconclusive, are often limited to diabetic populations, and exhibit significant discordance. This variability points to genetic background, geographical location, lifestyle, environmental factors, comorbidities, co-treatments/treatment history, age, and sex as potential confounding factors biasing clinical trial results. Additionally, the lack of standardized disease monitoring criteria and treatment regimens further complicate study outcomes, pointing to the urgent need of standardized clinical interventions for future large-scale investigations. To date, a dose ranging from 500 mg/day to 2000 mg/day has been adopted by most clinical trials [72,73,128], but synergism and/or interaction with other age-related drugs has been poorly addressed.

It is possible that the use of biomarkers for patient stratification and an unbiased machine learning algorithm will facilitate these efforts. 

### 3.2. Parkinson’s Disease

Parkinson’s disease (PD), one of the most common age-related neurodegenerative disorders, has a significant increase in incidence with aging [129]. Its pathological hallmarks, including the progressive degeneration of dopaminergic neurons in the *substantia nigra pars compacta*, as well as the presence of Lewy bodies composed of misfolded α-synuclein (SNCA) protein, lead to both motor (tremor, muscle rigidity, bradykinesia, and postural instability) and non-motor (including apathy, anhedonia, depression, and cognitive dysfunction) symptoms [130]. While the exact pathogenic mechanisms remain elusive, a growing body of evidence highlights the key role of OS, mitochondrial dysfunction, neuroinflammation, dysregulated autophagy, and aging in PD pathogenesis [131,132]. To date, there are no disease-modifying therapies for PD; the current pharmacological interventions focus on symptom alleviation rather than slowing down or preventing neuronal death progression [133]. Consequently, the global rise in the elderly population emphasizes the urgent need to explore new therapeutic approaches.

To date, there is evidence that mice with T2DM may have dopaminergic neurodegeneration [134] and patients with diabetes mellitus have an increased PD risk [135]. In this context, metformin may offer potential neuroprotective effects against PD development and progression. This hypothesis is corroborated by several in vitro and in vivo studies that document the encouraging impact of metformin in PD pathogenesis (Table 2) [71,136,137,138,139,140,141,142,143,144,145,146,147,148,149,150,151,152,153,154].

**Figure 3:**
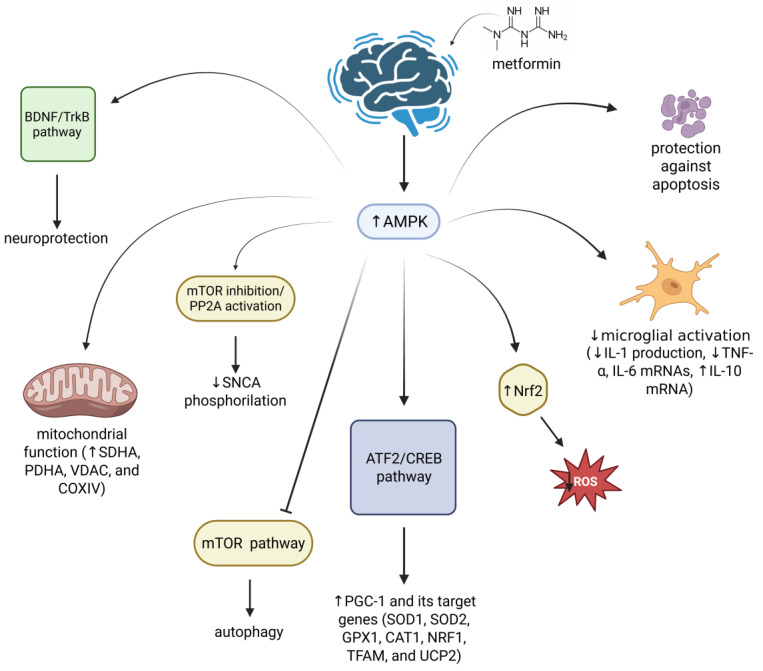
Potential mechanisms of action of metformin in PD. Abbreviations: AMPK: 5′ AMP-activated protein kinase; ATF2: activating transcription factor 2; BDNF: brain-derived neurotrophic factor; CAT1: cationic amino acid transporter-1; COXIV: cytochrome C oxidase subunit IV; CREB: cAMP response element-binding protein; GPX1: glutathione peroxidase-1; IL-1: interleukin 1; IL-6: interleukin 6; IL-10: interleukin 10; mTOR: kinase mechanistic target of rapamycin; NRF1: nuclear respiratory factor-1; Nrf2: nuclear factor erythroid 2-related factor 2; PDHA: pyruvate dehydrogenase; PGC-1α: peroxisome proliferator-activated receptor-gamma coactivator-1alpha; PP2A: protein phosphatase 2A; ROS: reactive oxygen species; SDHA: succinate dehydrogenase complex, subunit A; SNCA: alpha synuclein; SOD1: superoxide dismutase 1; SOD2: superoxide dismutase2; TFAM: mitochondrial transcription factor A; TNF-α: tumor necrosis factor alpha; TrkB: tyrosine receptor kinase B; UCP2: uncoupling protein-2; VDAC: voltage-dependent anion channel (Created with Biorender).

In vitro, metformin has been found to modulate various cellular pathways involved in PD pathogenesis (Figure 3). By regulating AMPK activity, metformin influences several subsequent pathways involved in autophagy, mitochondrial quality control (by regulating mitochondrial complex I activity, succinate dehydrogenase complex subunit A, pyruvate dehydrogenase, and voltage-dependent anion channel), and antioxidant processes (by increasing SOD1, SOD2, glutathione peroxidase-1, cationic amino acid transporter-1, nuclear respiratory factor-1, mitochondrial transcription factor A, and uncoupling protein-2), all dysregulated in PD [155]. As mentioned above, one of PD’s hallmarks is the presence of SNCA protein aggregates, whose formation is due to several post-translational modifications, including phosphorylation at Ser129, Ser87, and Tyr125 [156]. Of note, the protein phosphatase 2A (PP2A), the primary SNCA phosphatase, is dysregulated in the *post mortem* brain of PD patients [157]. Interestingly, metformin has been observed to activate PP2A and thus reduce SNCA phosphorylation by suppressing mTOR signalling, through AMPK-dependent and -independent mechanisms (in SH-SY5Y and HeLa cellular models, respectively) [145]. The presence of SNCA aggregation has been associated with impaired autophagy, a biological process crucial for degrading aggregated proteins [158]. In this regard, the pro-autophagic activity of metformin has been well documented [159]. Indeed, Lu and coworkers demonstrated the protective role of metformin on dopaminergic neurons via activation of the AMPK-autophagy pathway [140].

The potential role of metformin in counteracting mitochondrial dysfunction and OS, both key features of PD, has also been extensively investigated [136,138,140,142,160,161]. Interestingly, metformin has been reported to inhibit mitochondrial complex I activity, which is one of the major sources of superoxide and other ROS [162]. Specifically, Lu and coworkers demonstrated the ability of metformin to reduce the number of dysfunctional mitochondria and ROS production in 1-methyl-4-phenylpyridinium ion (MPP^+^)-treated SH-SY5Y in an AMPK-dependent manner, thus suggesting its neuroprotective effects [140]. Furthermore, another study underlined metformin’s capability of upregulating the expression of several mitochondrial proteins, including succinate dehydrogenase complex subunit A, pyruvate dehydrogenase, and voltage-dependent anion channel, in SH-SY5Y cells in a dose-dependent manner [142]. This mechanism appears to be AMPK-independent and involves the activation of the transcription factor 2 (ATF2)/CREB pathway, which induces the upregulation of the PGC-1α. Of note, PGC-1α is a transcriptional cofactor downregulated in PD brains and crucial for mitochondrial biogenesis and antioxidant defence [142,163]. In turn, the activation of this pathway results in the expression of PGC1α target genes, such as SOD1, SOD2, glutathione peroxidase-1, cationic amino acid transporter-1, nuclear respiratory factor-1, mitochondrial transcription factor A, and uncoupling protein-2 [142], thus suggesting metformin’s potential beneficial effects. In line with this study, Katila and colleagues revealed the neuroprotective effects of metformin in SH-SY5Y rotenone-treated cells. In detail, metformin protects these cells from rotenone-mediated death via reduction of ROS levels and restoration of mitochondrial function, by upregulating the expression of PGC-1α, as well as some antioxidant molecules and pathways (i.e., GSH, SOD, Nrf2) [150]. Nevertheless, it is worth noting the adverse effects of metformin in the MPP^+^-N27 dopaminergic cell line derived from rat ventral mesencephalon [138]. Indeed, the authors observed that the increase in ROS production and the reduction in ATP levels were aggravated when this PD cellular model was treated with 100 μM of MPP^+^ combined with metformin [138].

Another key process with a pivotal role in PD pathogenesis is neuroinflammation mediated by microglia [164]. In this context, metformin has been shown to partially reduce the mRNA of the pro-inflammatory cytokine IL-1β in BV2 cells treated with lipopolysaccharide (LPS) [136]. Despite these improvements, the authors did not find any alteration in mRNA levels of iNOS and TNF-α.

Based on these in vitro studies, which revealed the ability of metformin to modulate several key features of PD, the potential neuroprotective effects of this drug have been asserted. However, the controversial findings underline the need for further studies to better understand the molecular mechanisms of metformin and its possible use to counteract/prevent neurodegeneration.

In in vivo studies, metformin has been reported to prevent the degeneration of dopaminergic neurons (by upregulating BDNF and activating its downstream signalling pathways, including AKT, extracellular regulated protein kinase 1/2, GSK-3β, CREB); reduce neuroinflammation (lower values of TNF-α, IL-1β, IL-6, iNOS) and OS (by increasing Nrf2, SOD, catalase, GSH, thioredoxin, and HO-1, as well as *sod3*); and regulate autophagy (by suppressing the mTOR signalling pathway), SNCA phosphorylation (by suppressing the mTOR signalling pathway), and mitochondrial function (by increasing SDHA, PDHA, VDAC, and COXIV proteins). These mechanisms are summarized in Figure 2 (Figure 3; Table 2).

Regarding the behavioural deficits, metformin has been shown to ameliorate locomotor and muscular impairment in various PD animal models, including 1-methyl-4-phenyl-1,2,3,6-tetrahydropyridine (MPTP)-treated mice, 6-hydroxydopamine (6-OHDA)-lesioned mice, and rotenone mouse models [137,140,142,143,146,147]. However, other studies revealed conflicting results [139,149]. For example, Adedeji and coworkers found no effect of metformin on motor coordination, observing only a reduced duration in catalepsy score in a haloperidol-induced PD mouse model [139]. This discrepancy might be partially explained by the huge variability of animal models and metformin doses used across different studies.

As mentioned before, another hallmark of PD is the progressive loss of dopaminergic neurons and the consequent decrease in dopamine content within the brain [165]. While some studies have underlined the ability of metformin to prevent dopaminergic cell loss [137,140,141,142,143,144,147,148,149], others have reported contrasting results [136,138,146]. For instance, Patil and coauthors reported significant protection of tyrosine hydroxylase-positive cells, as well as increased levels of BDNF (a neurotrophic factor with neuroprotective and neurorestorative effects on dopaminergic neurons), in MPTP mice treated with metformin compared to the MPTP-only group [137,166]. Similarly, other works revealed the upregulation of BDNF and the activation of its downstream signalling pathways (AKT, extracellular regulated protein kinase 1/2, GSK-3β, CREB) in MPTP and 6-OHDA mice treated with metformin [143,146]. Conversely, other experiments performed on the LPS-induced rat neuroinflammation model and on MPTP-treated mice indicated that not only metformin failed to protect dopaminergic neurons from death, but also exacerbated neuronal damage [136,138]. Notably, MPTP and metformin may have an additive inhibitory impact on mitochondrial complex I, resulting in reduced ATP levels. This may explain the detrimental effect of the drug in neurons, which are particularly sensitive to complex I inhibition and get easily damaged [138]. Moreover, the neuroprotective efficacy of metformin could be influenced by the dosage and the treatment scheme. Nevertheless, other experiments have documented increased dopamine levels [140,143] and upregulation of the tyrosine hydroxylase cat2 gene (necessary for dopamine synthesis) following metformin administration [148].

Some studies have also reported metformin’s ability to prevent SNCA phosphorylation and aggregation [140,143,145,148,149]. In this context, metformin has been observed to lower phospho-Ser129 SNCA levels in the brain of mice [145] and attenuate SNCA accumulation in MPTP-plus-probenecid PD mice, through a mechanism involving AMPK phosphorylation and autophagy [143].

Neuroinflammation and OS are other two key hallmarks of PD [167]. In this context, a substantial body of evidence highlights the promising anti-inflammatory and antioxidant effects of metformin, possibly contributing to neuroprotection [136,137,138,139,140,147,148,149]. Of note, this drug has been demonstrated to reduce microglial activation and the consequent release of pro-inflammatory markers (mRNA and protein), including TNF-α, IL-1β, IL-6, iNOS. Simultaneous increase of anti-inflammatory cytokines, such as IL-10, was also observed [136,138,140]. Metformin can then attenuate lipid peroxidation, MDA and nitric oxide production, and increase the expression of the OS markers Nrf2, SOD, catalase (CAT), GSH, thioredoxin, and HO-1 in different PD animal models [137,139,147,148,149].

Despite the limited number of in vivo studies and some reported inconsistencies, substantial evidence supports the potential neuroprotective effects of metformin in PD neuropathology. However, further research is crucial to confirm these promising results and better clarify the possible role of metformin in managing this complex disease.

While preclinical studies have largely shown the beneficial effects of metformin in PD, clinical studies remain scarce. Even when conducted, those have primarily focused on the impact of metformin intake on PD risk in patients with T2DM, yielding conflicting results [71,151,152,153,154]. For instance, Wahlqvist and coauthors performed a cohort study investigating the potential effects of oral antihyperglycemic agents (OAAs), namely, sulfonylureas and metformin, on PD development. In detail, they found an increased PD risk in T2DM patients treated solely with sulfonylureas compared to those without OAAs therapy. Conversely, metformin alone, or in combination with sulfonylureas, did not affect PD risk [151]. Similarly, another study reported a reduced PD risk in elderly T2DM patients associated with prolonged (>4 years) metformin treatment [71]. In contrast, Kuan and colleagues found a higher PD development risk in the metformin-treated group in a retrospective cohort study involving 4651 T2DM patients treated with metformin and 4651 T2DM patients without metformin therapy [152]. These contradictory findings may be partially explained by the possible synergistic neuroprotective effects of metformin when combined with other anti-diabetic drugs with a different mechanism of action. Supporting this, Brakedal and coworkers reported a reduced risk of PD development with glitazone therapy compared to metformin-only treatment [153]. Lastly, a cross-sectional study found a dose–response relationship between metformin use and PD risk in T2DM patients. In detail, low dosage and intensity of metformin use were associated with a reduced probability of developing PD, while higher dosage and intensity showed no neuroprotective effect [154].

One key side effect to consider in the context of metformin use in PD therapy is the risk of developing vitamin B12 deficiency in diabetic patients treated with high metformin doses [168]. Given the association between low vitamin B12 levels and PD [169], it is plausible that high doses of metformin could increase PD risk by decreasing serum vitamin B12 levels [152]. Besides the dose, it is crucial to highlight that prolonged use of metformin also leads to vitamin B12 and folate deficiency together with higher homocysteine levels. These irregularities are linked with the risk of cognitive impairment and peripheral neuropathy [170,171]. Of note, homocysteine induces OS, neuronal apoptosis, and DNA damage [172]. Epidemiological studies revealed that hyperhomocysteinemia was associated with developing neurodegenerative disorders, including PD [173,174]. In this regard, some studies reported that higher plasma homocysteine levels are associated with poorer clinical outcomes and greater motor severity in PD patients [172,175]. In addition, a prospective study of 680 PD patients showed that low vitamin B12 levels at baseline were linked to greater worsening of motor symptoms in the early stages of the disease [176]. Notably, low vitamin B12 levels in patients with PD are associated with cholinergic transmission dysfunction and motor severity [175]. Therefore, metformin-induced vitamin B12 and folate deficiency with increased homocysteine levels could be potential links in the development and progression of PD. Thus, supplementation with folate and B12 in T2DM patients on metformin therapy might be a preventive measure to prevent cognitive impairment and PD development.

**Table 2:** Preclinical and clinical studies of metformin therapy in PD.

Preclinical Studies			
Model	Metformin Dose/Concentration and Duration	Treatment Effects	Reference
In vitro. MPP^+^-treated SH-SY5Y cells In vivo. MPTP/p PD mouse model	2 mM for 1 h 5 mg/mL in drinking water for 5 weeks	Protection against apoptosis ↓ the proportion of dysfunctional mitochondria and ROS generation Improvement in motor deficits ↑ dopamine levels ↓ DA neuron degeneration and SNCA accumulation ↓ NLRP3 inflammasome activity, ↓ IL-1 production, ↓ TNF-α, IL-6 mRNAs, ↑ IL-10 mRNA levels, no effect on IL-4 and TGF-β mRNA levels	[140]
In vitro. Tetracycline-treated SH-SY5Y cells In vitro. Transfected human SNCA HeLa cells In vivo. WT C57BL/6 mice	0.5, 1.0, or 2.5 mM for 16 h or 24 h 0.5, 1.0, or 2.5 mM for 16 h 5 g/kg in food pellets for 1 month or 5 g/L in drinking water for 6 months	↓ phospho-Ser129 SNCA levels ↓ phospho-Ser129 SNCA levels ↓ phosphorylated SNCA protein	[145]
In vitro. MPP^+^-treated SH-SY5Y cells In vivo. MPTP PD mouse model	0.1, 0.25, or 0.5 mM for 48 h 200 or 400 mg/kg in drinking water for 14 days	↑ mitochondrial marker proteins (SDHA, PDHA, VDAC, and HSP60) ↑ PGC-1α expression ↑ SOD1, SOD2, GPX1, CAT1, NRF1, TFAM, and UCP2 mRNAs ↑ mitochondrial marker proteins (SDHA, PDHA, VDAC, and COXIV) ↑ PGC-1α expression Protection of dopaminergic neurons Improvement in motor behaviour	[142]
In vitro. BV2 cells treated with LPS In vitro. BV2 cells treated with IL-4 In vivo. LPS-induced PD rat model	1 mM for 12 h Two daily oral doses (150 mg/kg) dissolved in tap water for 7 days	↓ microglial activation (↓ IL-1β mRNA levels, no effect on iNOS and TNF-α mRNAs) ↓ ROS production ↓ NLRP3 inflammasome activation ↓ microglial activation (↓ arginase mRNA levels, no effect on IL-10 mRNA levels) ↓ number of activated microglial cells ↓ TNF-α, IL-1β, and IL-6 mRNA levels; ↑ MCP-1, CD200, and CX3CR1 mRNA levels No effect on dopaminergic neuronal loss protection	[136]
In vitro. SH-SY5Y cells treated with rotenone	10 μM, 100 μM, 1 mM, or 10 mM for 2 h, 3 h, 6 h	↓ cellular death and 3/7 caspase activation ↓ ROS production ↑ GSH and SOD levels Upregulation of Nrf2/HO-1 pathway ↑ PGC-1α levels	[150]
In vitro. MPP^+^-treated SH-SY5Y cells In vivo. MPTP PD mouse model	0.5 mM for 4 h Once daily intraperitoneal injection (200 mg/kg) for 7 days	Neuroprotection is partially mediated by the BDNF/TrkB signaling pathway ↓ Caspase-3 ↑ Dopamine and DOPAC levels Improvement in motor deficits ↓ Astroglial activation ↓ SNCA levels ↑ BDNF, AKT and ERK1/2	[143]
In vitro. MPP^+^ -N27 dopaminergic cell line derived from rat ventral mesencephalon In vivo. MPTP PD mouse model	0.1 mM or 1 mM for 24 h Two daily doses (150 mg/kg) for 7 days	↓ ATP production ↑ ROS production ↓ TNF-α, IL-1β, and iNOS mRNAs levels No effect on dopaminergic neuronal loss ↓ DOPAC	[138]
In vivo. 6-OHDA PD mouse model	Once daily oral doses (100 mg/kg or 200 mg/kg) for 4 weeks	Improved motor impairments No protective effect against dopaminergic cell death ↑ AMPK, AKT, GSK3b, CREB phosphorylation, and BDNF levels ↓ Astrocyte activation	[146]
In vivo. MPTP PD mouse model	500 mg/kg orally for 21 days	Improved locomotor and muscular activities ↑ SOD, CAT, and GSH levels ↓ Lipid peroxidation ↑ BDNF levels	[137]
In vivo. Haloperidol-induced catalepsy PD mouse model	Once daily oral doses (20, 50, or 100 mg/kg) for 21 days	No effect on motor coordination, but improvement in memory deficit ↓ Duration of catalepsy score ↓ MDA and nitric oxide levels, and ↑ GSH and CAT activity No effect on SOD activity	[139]
In vivo. MPTP AMPK WT and KO PD mouse model	100 mg/kg dissolved in water for 27 days	Attenuation in the loss of neuron number and volume, as well as an increase in gliosis ↓ DOPAC:DA ratio	[141]
In vivo. MDMA PD mouse model	200–400 mg/kg orally 11 h intervals for 48 h and 7 days	Attenuation of TH-positive neuronal loss	[144]
In vivo. Rotenone PD mouse model	Once daily oral doses (100 or 200 mg/kg) for 18 days administered through a gastric gavage tube	Improvement in animals’ motor function ↓ MDA, ↑ GSH, HO-1, and dopamine levels ↑ Nrf2, thioredoxin, AMPK, and FOXO3 mRNA levels ↑ Nrf2, HO-1, AMPK, FOXO3, and thioredoxin protein expression ↓ Cleaved-caspase 3 and VEGF levels ↑ the number of TH-positive neurons	[147]
In vivo. Rotenone PD mouse model	Once daily intraperitoneal injection (300 mg/kg) for 10 days	No difference in motor behaviour Partial attenuation of dopaminergic neuronal loss ↓ Caspase-3 ↓ SNCA accumulation ↓ 4-HNE and MDA levels	[149]
In vivo. 6-OHDA PD *C. elegans* strain BZ555 model	Oral doses (5 mM or 10 mM) for 72 h	Increase in lifespan ↓ Dopaminergic neurons degeneration ↓ SNCA aggregation ↑ TH gene *cat3* and antioxidant gene *sod3*	[148]
In vivo. RNAi-mediated knockdown of *C. elegans bcat-1* PD model	50 μM for 5 days	↑ the number of dopaminergic cell bodies Improvement in neurite morphologies of dopaminergic neurons Restoration of normal mitochondrial activity levels	[177]
**Clinical Studies**			
**Study Method**	**Subjects**	**Treatment Effects**	**Reference**
Retrospective cohort study	n = 1879 T2DM patients treated with metformin only for 11 years n = 3431 T2DM patients treated with sulfonylureas only for 11 years n = 6420 T2DM patients treated with metformin + sulfonylureas for 11 years	Increased PD risk in T2DM patients treated with sulfonylureas (HR = 1.57, 95% CI = 1.15–2.13) No effect on PD risk in T2DM patients treated with metformin (HR = 0.95, 95% CI = 0.53–1.71) and with sulfonylureas plus metformin (HR = 0.78, 95% CI = 0.61–1.01)	[151]
Retrospective cohort study	n = 4651 T2DM patients treated with metformin for at least 90 days n = 4651 T2DM patients with no metformin therapy	Higher PD risk (HR = 2.27, 95% CI = 1.68–3.07) in the metformin group Higher PD risk in patients with T2DM receiving metformin therapy for 300–399 days (aHR = 2.20, 95% CI = 1.47–3.28) and ≥400 days (aHR = 4.49, 95% CI = 3.06–6.58) PD risk increased from 1.58 (95% CI = 1.02–2.44) in patients receiving average doses of metformin ≤ 130 g per year to 3.54 in patients receiving average doses of >385 g per year (95% CI = 2.41–5.20)	[152]
Retrospective cohort study	n = 8396 T2DM patients treated with glitazones for 10 years n = 94,349 T2DM patients treated with metformin for 10 years	Lower PD risk in patients with T2DM receiving glitazone drug compared to the metformin group (HR = 0.72, 95% CI = 0.54–0.94)	[153]
Cross-sectional study	n = 384,716 T2DM patients treated with metformin	Higher ORs for PD in patients with T2DM with increased cDDD of metformin after 3 years: <300 (OR = 0.88, 95% CI = 0.83–0.94), 300–500 (OR = 1.09, 95% CI = 0.72–1.65), and >500 (OR = 2.59, 95% CI = 0.83–8.03) g per year Higher ORs for PD in patients with T2DM with increased intensity of metformin use (DDD/month) after 3 years: <10 (OR = 0.87, 95% CI = 0.81–0.93), 10–25 (OR = 0.92, 95% CI = 0.83–1.02), and ≥25 (OR = 1.17, 95% CI = 0.80–1.72) g per month Higher ORs for PD in patients with T2DM with increased cDDD of metformin after 5 years: <300 (OR = 0.94, 95% CI = 0.90–0.98), 300–500 (OR = 1.01, 95% CI = 0.75–1.35), and >500 (OR = 1.24, 95% CI = 0.40–3.83) g per year Higher ORs for PD in patients with T2DM with increased intensity of metformin use (DDD/month) after 5 years: <10 (OR = 0.93, 95% CI = 0.89–0.98), 10–25 (OR = 0.97, 95% CI = 0.90–1.04), and ≥25 (OR = 1.02, 95% CI = 0.77–1.35) g per month	[154]
Retrospective longitudinal cohort study	n = 2756 T2DM patients without metformin treatment n = 849 T2DM patients treated with metformin for less than 1 year n = 513 T2DM patients treated with metformin for 1–2 years n = 710 T2DM patients treated with metformin for 2–4 years n = 700 T2DM patients treated with metformin for more than 4 years	Lower risk of PD in elderly patients with T2DM after more than 4 years of metformin exposure (aHR = 0.04, 95% CI 0.00–0.37)	[71]

Abbreviations: aHR: adjust hazard ratio; AKT: protein kinase B; AMPK: AMP-activated protein kinase; ATP: adenosine triphosphate; BDNF: brain-derived neurotrophic factor; CAT: catalase; CAT1: cationic amino acid transporter-1; cDDD: cumulative DDD; CI: confidence interval; COXIV: cytochrome C oxidase subunit IV; CREB: cAMP response element-binding protein; CX3CR1: C-X3-C motif chemokine receptor 1; DA: dopamine; DDD: defined daily doses; DOPAC: 3,4-dihydroxyphenylacetic acid; Erk1/2: extracellular signal-regulated kinase 1/2; FOXO3: forkhead box O3; GPX1: glutathione peroxidase-1; GSH: glutathione; GSK3b: glycogen synthase kinase-3 beta; 4-HNE: 4-hydroxynonenal; HO-1: heme oxygenase 1; HR: hazard ratio; HSP60: heat shock protein 60; IL-1β: interleukin 1β; IL-4: interleukin 4; IL-6: interleukin 6; IL-10: interleukin 10; iNOS: nitric oxide synthase; KO: knockout; LPS: lipopolysaccharide; MCP-1: monocyte chemoattractant protein 1; MDA: malondialdehyde; MDMA: 3,4-methylenedioxymethamphetamine; MPP^+^: 1-methyl-4-phenylpyridinium; MPTP/p: MPTP/probenecid; MPTP: 1-methyl-4-phenyl-1,2,3,6-tetrahydropyridine; NLRP3: NLR family pyrin domain containing 3; NRF1: nuclear respiratory factor-1; Nrf2: nuclear factor erythroid 2-related factor 2; 6-OHDA: 6-hydroxydopamine; OR: odd ratio; PD: Parkinson’s disease; PDHA: pyruvate dehydrogenase; PGC-1α: peroxisome proliferator-activated receptor-gamma coactivator-1alpha; ROS: reactive oxygen species; SDHA: succinate dehydrogenase complex, subunit A; SNCA: alpha synuclein; SOD: superoxide dismutase; SOD1: superoxide dismutase 1; SOD2: superoxide dismutase2; T2DM: type 2 diabetes mellitus; TFAM: mitochondrial transcription factor A; TGF-β: transforming growth factor beta; TH: tyrosine hydroxylase; TNF-α: tumor necrosis factor alpha; TrkB: tyrosine receptor kinase B; UCP2: uncoupling protein-2; VDAC: voltage-dependent anion channel; VEGF: vascular endothelial growth factor; WT: wild type; ↑ increase; ↓ decrease.

Based on this evidence, it is noticeable that metformin could have beneficial and harmful effects in PD depending on the duration and dose. Further clinical studies are mandatory to better clarify the effects of metformin on PD risk. Moreover, as existing studies have focused on T2DM patients without PD, it is also crucial to expand the investigations including patients already diagnosed with this neurodegenerative disease in order to assess whether metformin may protect against PD progression.

### 3.3. Cardiovascular Disease

Cardiovascular disease (CVD) is a broad term encompassing a range of disorders affecting the heart and blood vessels, including coronary artery disease (CAD), cerebrovascular diseases, peripheral artery disease, and aortic atherosclerosis. CVD remains a leading cause of death worldwide, despite being largely preventable. Also known as coronary heart disease, CAD, the most prevalent type of CVD, results from decreased myocardial perfusion that causes angina (chest pain due to reduced blood flow to the heart), myocardial infarction (occurring when a blood vessel supplying the heart is abruptly blocked, potentially damaging the heart muscle), and heart failure (HF; the heart’s inability to pump blood effectively, leading to fluid buildup in the lungs and breathing difficulties). Stroke is one of the most common cerebrovascular diseases and occurs when a blood vessel supplying the brain is either blocked (ischaemic stroke) or ruptures and bleeds (hemorrhagic stroke). Peripheral artery disease affects the limbs, while aortic atherosclerosis involves thoracic and abdominal aneurysms [178]. Abdominal aortic aneurysms (AAAs) are the most common type of aneurysm, and a “watch and wait” approach with regular surveillance is the standard of care for small aneurysms, as there are currently no effective drug treatments available to limit their growth [179]. Several risk factors lead to CVD, including smoking, hypertension, high cholesterol, high lipoproteins, obesity, and diabetes mellitus [180]. The treatments for CVD are diverse and tailored to the specific type of heart disease, encompassing lifestyle changes, medications (including anticoagulants, antiplatelet therapies, angiotensin-converting enzyme inhibitors, beta-blockers, calcium channel blockers, cholesterol-lowering medications, and diuretics), and/or medical procedures [181].

**Figure 4:**
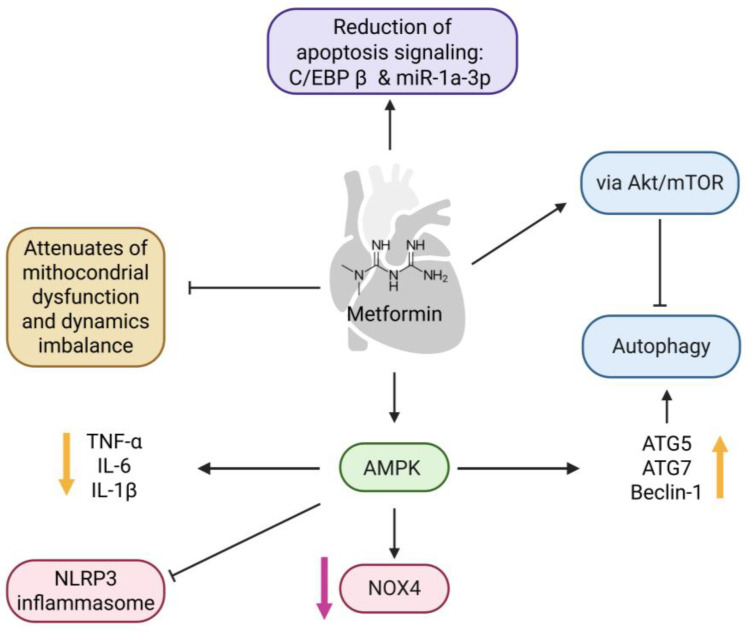
Potential mechanisms of action of metformin in CVD. Abbreviations: Akt: protein kinase B; AMPK: 5′ AMP-activated protein kinase; ATG5: autophagy related 5; ATG7: autophagy related 7; C/EBP β: CCAAT/enhancer-binding protein beta; IL-1β: interleukin 1beta; IL-6: interleukin 6; miR-1a-3p: microRNA-1 family; mTOR: kinase mechanistic target of rapamycin; NLRP3: NLR family pyrin domain containing 3; NOX4: NADPH oxidase 4; TNF-α: tumor necrosis factor alpha (Created with Biorender).

The beneficial effects of metformin have been demonstrated in various animal models of cardiovascular disorders, especially atherosclerosis, myocardial injury, and HF [6,182]. Recent studies have identified additional novel molecular targets contributing to metformin’s cardiovascular protection (see below). These findings provide crucial insights into the molecular mechanisms underlying metformin’s cardioprotective action, thereby expanding the foundation for further clinical trials to conclusively establish its efficacy in the management of specific cardiovascular disorders. In particular, some cardioprotective effects of metformin are mediated by AMPK (see Figure 4). AMPK kinase plays an important role in regulating myocardial energy metabolism and reducing I/R injury [183,184]. Of note, metformin can protect against apoptosis induced by cardiac I/R injury through AMPK activation. Metformin was shown to reduce the size of the infarct and inhibit cardiac fibrosis through the reduction of proinflammatory cytokines (TNF-α, IL-6, IL-1β) and the suppression of the activation of the NLR family pyrin domain containing 3 (NLRP3) inflammasome in isolated rat hearts subjected to I/R [185]. Concerning OS, metformin upregulates the phosphorylation of AMPK and decreases the *NOX4* gene expression, leading to the decrease in myocardial oxidative damage. This is accompanied with higher levels of antioxidant enzymes such as MnSOD which alleviate reperfusion injury [184]. Metformin has also been reported to protect cardiomyocytes against H_2_O_2_-induced apoptosis through the AMPK/CCAAT-enhancer-binding protein (C/EBP) beta/miR-1a-3p/grpq4 (β/miR-1a-3p/GRPQ4) pathway. In detail, miR-1a-3p follows protein GRP94, which results in the accumulation of unfolded or misfolded proteins, generating ER stress. C/EBP β directly induces the upregulation of miR-1a-3p by binding to its promoter. According to the research, metformin has a positive impact on AMPK and also decreases the levels of C/EBP β and miR-1a-3p, especially when compared to the control group in the study [186]. These results confirm the positive effect of metformin for the treatment of cardiac I/R injury by attenuating cell apoptosis. Figure 4 summarizes the different pathways through which metformin exerts cardioprotective effects in cardiomyocytes.

Preclinical and clinical findings on metformin’s cardiovascular protection are summarized in Table 3 [187,188,189,190,191,192,193,194,195,196,197,198,199,200,201,202,203,204,205,206,207,208,209].

Emerging preclinical studies have identified new metformin’s modes of action, including the activation of the AMPK/autophagy axis, the suppression of mitochondrial ROS via AMPK-independent pathway, the activation of sirtuin signaling, as well as autophagy-dependent pathway [187,188,189,190,191,192,193,194,195,196,197]. Concerning the first two mechanisms related to its atheroprotective role, some works performed on different animal models have shown that metformin attenuates atheromatous plaque formation, suppresses atherogenesis, improves autophagy-mediated cholesterol efflux in vascular smooth muscle cells-derived foam cells (a significant component of atherosclerotic lesions), and reduces glucose production [187,188,189,190]. Conversely, through the activation of the latter two mechanisms, metformin was reported to ameliorate myocardial damage, protect against cardiotoxicity and cardiac hypertrophy, and prevent cardiomyopathy [192,194,195,196]. However, despite these promising results, the studies are limited. Therefore, further preclinical investigations are necessary to confirm and better clarify the molecular mechanisms underlying metformin’s effects. Nevertheless, recent clinical studies have corroborated the atheroprotective effects of metformin. In this regard, several randomized controlled trials (RCTs) have demonstrated the ability of metformin to improve the metabolic status and reduce the progression of atherosclerosis in non-diabetic and diabetic patients with T1DM and T2DM [198,199,200,201,202,203]. This consistent positive evidence from both preclinical and clinical studies prompted the development of large-scale RCTs to investigate the atheroprotective effects of metformin in specific patient populations. The multicenter VA-IMPACT trial, involving over 7400 participants and currently in the recruiting phase, aims to evaluate the effects of metformin treatment on reducing mortality and cardiovascular morbidity in patients with pre-diabetes and established atherosclerotic cardiovascular disease, compared with placebo (NCT02915198). Furthermore, a few small-scale RCTs have also demonstrated positive effects of this drug on myocardial metabolic and functional parameters, even in patients with HF [204,205,206,207,208,209]. In this latter context, while the observational studies have been limited to patients with diabetes, the results suggest improved cardiovascular outcomes with metformin treatment [205,206,207]. Consequently, randomized trials are mandatory to further explore these findings in patients both with and without diabetes. These studies highlight the cardiovascular and metabolic benefits of metformin; however, large-scale RCTs, such as the VA-IMPACT trial, are needed to assess the cardiovascular outcomes of metformin use, particularly in patients at high risk of adverse cardiovascular events and HF. 

Regarding aneurysms, the current body of evidence concerning metformin is characterized by a significant divergence between observational and interventional data. A systematic review and meta-analysis of 11 articles, comprising over 110,000 patients, found that metformin use was associated with a statistically significant reduction in the annual progression of AAA diameter, with a weighted mean difference of −0.84 mm. The pooled data also suggested an association with a decrease in AAA-related events, such as rupture and mortality. Although promising, these findings are based on observational studies, which can show association but are prone to confounding factors. For instance, healthier patients may be more likely to be prescribed metformin, leading to a healthier cohort overall [210]. This is where the importance of the ongoing and future randomized controlled trials becomes clear. The completed, underpowered trial demonstrated no significant effect on aneurysm growth, but it could not definitively rule out a small, clinically relevant effect due to its small sample size. The ongoing, large-scale LIMIT (NCT04500756) and MAT trials are designed precisely to address this evidence gap. By enrolling a large cohort and following participants over a period of years in a placebo-controlled, blinded setting, these trials will provide the robust, high-level evidence necessary to confirm or refute the observational findings. A positive outcome would be a transformative event in vascular medicine, providing the first pharmacological treatment for a disease that currently has no drug therapy.

Observational data and specific trial findings have led to a well-established reputation of the drug for providing cardioprotection. However, a meticulous review of the literature reveals a fundamental paradox: compelling evidence from multiple large-scale studies and meta-analyses demonstrates a lack of a statistically significant effect on broad, composite CVD endpoints [211]. This ambiguity is not a sign of the drug’s ineffectiveness but rather a reflection of the intricate relationship between its mechanism of action, the heterogeneity of patient populations, and the statistical limitations of composite endpoints in clinical trials. This controversy is supported by data from a large meta-analysis performed by Shabil and coworkers [211], a critical re-evaluation of the foundational UK Prospective Diabetes Study (UKPDS) [212], and findings from trials in non-diabetic populations [213]. Furthermore, a recent meta-analysis of 22 studies, encompassing over 612,000 participants [211] found that metformin treatment may not have a clear, significant effect on composite CVD outcomes as a whole. Given these data, the authors concluded that metformin may not have a significant effect on a composite of events such as stroke, myocardial infarction (MI), heart failure (HF), and major adverse cardiovascular events (MACE) [211].

The hypothesis that metformin has direct cardioprotective properties, independent of its glucose-lowering effects, led to its investigation in non-diabetic populations. However, the results from these trials have been mixed and largely disappointing [213]. The GIPS-III trial, which studied metformin in patients with ST-segment elevation myocardial infarction who did not have diabetes, showed as “little or no effect”. This failure suggests that the direct cardioprotective effects alone may be insufficient to produce a statistically significant clinical outcome. It is the synergistic effect of these direct mechanisms combined with the systemic improvements of glycemic control (e.g., reduced inflammation, improved endothelial function, better lipid profiles) that likely drives the positive outcomes observed in studies of diabetic patients.

### 3.4. Age-Related Macular Degeneration

Age-related macular degeneration (AMD) represents the primary cause of vision loss among adults older than 65 years and can result in permanent blindness. AMD causes pathological alterations in the deeper retinal layers of the macula and the adjacent vasculature, resulting in impaired central vision [214,215,216].

In 2020, approximately 200 million individuals worldwide were affected by AMD, and this number is projected to increase to around 288 million by 2040. The prevalence of AMD is expected to continue rising in parallel with global population aging, as aging is the main risk factor for AMD [217].

AMD is clinically categorized into two primary types: dry (atrophic) and wet (neovascular). Dry AMD is defined by the lack of serum or blood leakage and is often identified by the formation of drusen, retinal deposits that act as an early sign of the disease’s dry stage. Dry AMD is the most prevalent form and can progress to wet AMD, wherein the formation of choroidal neovascular membranes leads to bleeding and exudation within the retina, causing substantial vision loss [217,218]. Therapeutic options for AMD remain limited, particularly for the dry form. While some vitamin formulations have demonstrated efficacy in slowing the progression of dry AMD, there are currently no available treatments capable of reversing or curing this condition. Evidence suggests that lifestyle modifications, such as smoking cessation, regular physical activity, and maintaining a nutritious diet, may reduce the risk of developing AMD or slow its progression. For wet AMD, anti-vascular endothelial growth factor (anti-VEGF) agents represent the primary therapeutic approach by inhibiting VEGF, a protein responsible for promoting abnormal blood vessel growth in the retina, thereby preventing further macular damage and vision loss. However, these treatments are invasive, costly, and cannot reverse existing retinal damage. Given the limitations of current therapies for AMD, prevention and novel therapeutic strategies are critical for reducing disease prevalence and improving patient outcomes [219,220]. One promising approach is drug repurposing, which involves identifying new clinical applications for established medications. Metformin, a well-tolerated agent with an extensive safety record in the treatment of T2DM as previously underscored, has attracted growing interest as a potential candidate for AMD management due to its diverse pharmacological properties, including antioxidants, anti-inflammatory, antiangiogenic, and antifibrotic effects [221,222,223,224]. These mechanisms are relevant to pathways implicated in AMD pathogenesis. Furthermore, metformin has been postulated to delay aging and mitigate age-related diseases, making it particularly appealing for conditions such as AMD [225]. Preliminary research indicates that metformin may modulate cellular processes involved in AMD progression, highlighting its potential as a therapeutic option to slow or prevent the onset of this vision-threatening disorder, specifically in the elderly [225,226].

To the best of our knowledge, only one randomized clinical trial has evaluated the effect of metformin on AMD in patients without DM. This study enrolled 66 participants aged 55 years or older without DM, all diagnosed with geographic atrophy (GA), an advanced form of dry AMD, in one or both eyes. Among these, 34 participants (57 eyes) were allocated to the observation group, whereas 32 people (53 eyes) were given oral metformin. The main outcome was the annualized rate of GA advancement, which showed no significant difference between the metformin and observation groups. It should be highlighted that the restricted sample size may have diminished the statistical power to identify a possible treatment effect [227]. Despite GA development being a late-stage AMD [228], metformin may still offer benefits by affecting early disease processes, including the onset and progression of hypertransmission abnormalities, drusen accumulation, or neovascularization. In this regard, although no additional randomized clinical trials have been reported, several cohort studies, case–control studies, and case reports have investigated the association between metformin use and the development or progression of AMD. However, only a limited number of these studies have specifically examined the effect of metformin on AMD development in elderly individuals without T2DM. For instance, Aggarwal et al. [214], in a case–control study, reported that metformin use was associated with a 17% reduction in the odds of developing AMD among participants with a mean age of 74 years. Importantly, this association did not appear to be dose-dependent. Specifically, for any form of AMD, a risk reduction was observed across all groups. However, this protective effect was limited to participants in the two lowest quartiles of cumulative metformin use for dry AMD. Furthermore, in a case–control study involving patients aged 60 years and older with newly diagnosed GA, metformin use was evaluated in a subgroup without DM. In this subgroup, metformin use was associated with a 47% reduction in the odds of developing GA (95% CI, 0.33–0.83) [229].

Nevertheless, it should be again underscored that, since metformin is primarily prescribed for diabetes, the majority of cohort, case–control, and cross-sectional studies have included patients with DM. This makes it challenging to distinguish the effects of metformin on AMD from the inherent risks associated with DM itself. Diabetes is a recognized risk factor for both forms of AMD due to its impact on the retinal-endothelial system. Thus, evaluating the effect of metformin on AMD in individuals with DM may introduce confounding, as any observed protective effect could be influenced by the underlying diabetic condition [230,231]. Table 4 [232,233,234,235,236,237,238,239] summarizes some of these recent studies. While current observational evidence suggests a potential protective role of metformin (especially low dose) in AMD, randomized clinical trials are necessary to confirm these findings and to establish the clinical relevance and optimal dosing of metformin as a preventive therapy for AMD.

As mentioned, metformin affects different cellular pathways and has shown beneficial effects in many age-related disorders. Its potential impact on AMD is believed to be mediated through several distinct molecular mechanisms. In this regard, metformin has been proposed to exert both anti-inflammatory and antioxidative properties [13,240] that may contribute to its therapeutic benefit in AMD. In an in vitro study utilizing hydrogen peroxide (H_2_O_2_)-cultured ARPE-19 cells (an immortalized cell line of human retinal pigment epithelial cells) as a model of AMD, metformin was shown to activate the Nrf2 pathway—a central regulator of cellular antioxidant defence—by promoting both nuclear translocation and DNA-binding activity of Nrf2, resulting in increased expression of downstream antioxidant enzymes [241].

In another study, ARPE-19 cells pre-treated with metformin showed significantly reduced mitochondrial damage following exposure to antimycin A (a highly effective inhibitor of cellular respiration), with metformin pre-treatment resulting in an 84.5% reduction in damage. These findings indicate that metformin possesses both anti-inflammatory and antioxidant properties and promotes mitophagy in human ARPE subjected to mitochondrial injury [242]. To more accurately simulate AMD in vitro, primary cultured RPE cells can be utilized. Accordingly, in vitro experiments using D407 cells (a human RPE cell line) showed that metformin treatment upregulated autophagy, as indicated by increased LC3B expression and decreased p62 levels. These changes are characteristic of enhanced autophagic activity. Indeed, inhibition of autophagy abrogated metformin-induced alterations in LC3B and p62, as well as its protective effects against H_2_O_2_-induced loss of cell viability and apoptosis. Similarly, knockdown of the essential autophagy genes Beclin1 and LC3B also prevented metformin’s protective effects, suggesting that metformin confers resistance to H_2_O_2_-induced oxidative damage in D407 cells primarily through the stimulation of autophagy [243].

Metformin has also been shown to exert anti-angiogenic effects. Several studies have demonstrated that metformin inhibits angiogenesis in human retinal vascular endothelial cells both in vitro and in vivo in a dose-dependent manner [244,245]. In a separate study utilizing a neovascular AMD mouse model, intravitreal administration of metformin significantly reduced new vessel growth in choroidal explants, again exhibiting a dose-dependent effect. Intravitreal metformin treatment also suppressed choroidal neovascularization (CNV) and decreased peripheral infiltration of IBA1+ macrophages/microglia. Furthermore, metformin downregulated the expression of genes associated with angiogenesis and inflammation, including DLL4 (delta-like canonical Notch ligand 4) and IBA1 in the choroid and retinal pigment epithelium, two key processes implicated in the progression of neovascular AMD [223]. However, it is crucial to underscore that while current observational studies indicate a potential protective effect of metformin against AMD, these findings predominantly originate from diabetic populations, which limits their direct applicability to non-diabetic individuals. As mentioned earlier, only one case–control study has examined metformin use in non-diabetic individuals, finding it to be associated with reduced odds of developing AMD. However, this association was not dose-dependent, with protective effects observed primarily at lower cumulative metformin intake, raising questions about consistency and biological plausibility [214]. Similar findings in diabetic populations suggest protective effects of metformin at lower doses, while higher doses yield mixed results [246].

Randomized clinical trials are essential to confirm these effects, establish optimal dosing, and evaluate metformin’s potential as a preventive or therapeutic agent for AMD, particularly in non-diabetic individuals. If proven effective, metformin could serve as an affordable and accessible adjunct for managing AMD, addressing the limited treatment options currently available.

Overall, metformin use in the elderly, including individuals without DM, may offer a promising approach for both early and late stages of AMD. Its proven efficacy in geriatrics, ease of administration, and favourable safety profile position it as an alternative option in AMD. Nonetheless, contraindications, especially at higher doses, require careful consideration in this population.

### 3.5. Osteoporosis

Osteoporosis (OP) is one of the most prevalent disorders affecting the elderly. It is characterized by a reduction in bone mineral density and a progressive deterioration of bone microarchitecture, which collectively result in increased bone fragility and a significantly elevated risk of fractures [247,248].

The prevalence of OP increases with age, making it a significant health concern among older adults [249]. It is predicted that one in three women and one in five men over 50 will experience osteoporotic fractures [250]. Notably, 20% of patients suffering from a pelvic fracture die within a year [251]. Further, bone fractures, especially hip fractures, significantly impair daily functioning, with only one-third of patients eventually requiring long-term care in nursing facilities [252].

OP is associated with numerous complications that can profoundly impact patients’ quality of life, increase the risk of hospitalization, long-term disability, and raise mortality rates. Moreover, OP imposes a significant financial burden on the healthcare system, with estimated annual costs ranging between $13–22 billion in the U.S. alone [250,253].

Globally, the burden of OP is estimated to range between 19.7% and 21.7% among individuals aged 50 years and older. Women exhibit higher prevalence rates primarily due to a significant decrease in estrogen levels after menopause, as estrogen is essential for preserving bone density and inhibiting bone loss [248,254,255]. Sex differences in metformin’s effects on bone health are most pronounced in postmenopausal women, where estrogen deficiency accelerates bone loss and alters bone remodeling dynamics. In this population, metformin may be associated with a lower risk of osteoporosis and higher bone mineral density (BMD), independent of diabetes status or obesity, with the effect being particularly significant in women compared to men [256,257]. Mendelian randomized clinical studies further support a causal relationship between metformin use and reduced osteoporosis risk, as well as increased BMD at the lumbar spine and femoral neck, with the protective effect most evident in women [257,258,259]. Estrogen-related mechanisms modulate the magnitude of metformin’s impact on bone health and fracture risk, with the greatest benefit observed in estrogen-deficient states such as postmenopause, but metformin’s actions are not dependent on estrogen itself. Estrogen deficiency amplifies oxidative stress and osteoclast activity, both of which are targeted by metformin’s actions. The drug’s efficacy appears greater in the context of low estrogen, as seen in postmenopausal women and animal models, suggesting that metformin’s bone-protective effects are at least partially estrogen-independent but may be enhanced by the altered bone microenvironment following menopause [257,260,261].

A higher prevalence of OP has also been observed in certain regions, with the condition being more common in developing countries compared to developed ones. This is partially due to factors such as poor dietary habits, limited access to healthcare, and other related risk factors [248,254,255].

Beyond its antidiabetic properties, emerging evidence suggests that metformin may also benefit bone health and OP, particularly in the elderly population [257,258]. These protective effects are mediated through several key mechanisms, among which AMPK is a central regulator of cellular energy homeostasis and plays a critical role in bone metabolism by influencing both osteoblast and osteoclast activity [262]. As an AMPK activator, metformin may exert significant effects on bone remodeling through multiple pathways, supported by findings from both in vitro and in vivo studies [263].

#### 3.5.1. Effects on Osteoblasts

Osteoblasts are responsible for osteogenesis, including matrix synthesis and mineralization. Metformin seems to promote osteoblast differentiation from mesenchymal stem cells (MSCs) via AMPK activation, which regulates several downstream signaling pathways [263], as briefly described below.

The activation of AMPK promotes the phosphorylation and functional activity of runt-related transcription factor 2 (Runx2), which plays a central role in regulating osteoblast differentiation. Consequently, this process drives the upregulation of osteogenic markers, including alkaline phosphatase (ALP), osteocalcin (OCN), and bone morphogenetic proteins (BMPs) [264]. An in vitro study using MSCs derived from mouse cranial bones showed that metformin significantly upregulated Runx2 expression and promoted osteogenesis via AMPK activation [265]. Similarly, Wang et al. [263] reported that inhibition of liver kinase B1 (LKB1), an upstream activator of AMPK, reversed metformin-induced Runx2 expression and suppressed osteogenic differentiation in MSCs.

The canonical Wnt signaling pathway plays a pivotal role in regulating the proliferation and differentiation of MSCs and osteoblast progenitor cells and also influences osteoclast-mediated bone resorption. These combined effects contribute to bone formation and are crucial for sustaining bone homeostasis. Furthermore, other pathways, including the noncanonical Wnt, Janus kinase (JAK)/signal transducer and activator of transcription (STAT), and Hedgehog (Hh) pathways, interact with the Wnt/β-catenin signaling pathway through complex crosstalk mechanisms, collectively supporting the regulation of bone homeostasis. Given its central role in skeletal biology, the Wnt signaling pathway has emerged as a promising target for the development of novel therapeutics aimed at enhancing bone synthesis [266]. Whitin this context, metformin exerts its osteogenic effects, in part, by inhibiting glycogen synthase kinase-3β (GSK3β), a key negative regulator of the Wnt/β-catenin signaling pathway. Indeed, by suppressing GSK3β activity, metformin stabilizes β-catenin in the cytoplasm, preventing its degradation. This stabilization facilitates, in turn, the accumulation of β-catenin, enabling its translocation into the nucleus, where it interacts with transcription factors, including T-cell factor/lymphoid enhancer factor (TCF/LEF). These interactions promote the expression of osteogenic genes, such as those encoding ALP, OCN, and BMP, which are critical for osteoblast differentiation and function [267,268]. The activation of the Wnt/β-catenin pathway by metformin underscores its potential as a therapeutic agent for promoting bone formation and maintaining skeletal health [268].

Metformin also stimulates the expression of endothelial nitric oxide synthase (eNOS), a key enzyme involved in nitric oxide (NO) production through AMPK activation. NO plays a critical role in bone metabolism by promoting osteoblast proliferation, differentiation, and mineralization while simultaneously inhibiting osteoclast activity. Gu et al. demonstrated that low concentrations of metformin (0.05 mM) significantly increase osteogenesis in human villous MSCs by upregulating eNOS expression. This effect was accompanied by a suppression of adipocyte differentiation, suggesting that metformin not only favors osteogenic pathways but also inhibits alternative differentiation routes, such as adipogenesis [269]. The AMPK/eNOS/NO pathway appears to be central in mediating metformin’s osteogenic effects. Indeed, nitric oxide produced via eNOS activation has been shown to enhance the expression of osteogenic markers, including ALP, OCN, and BMPs, which, as mentioned, are essential for osteoblast function and matrix mineralization [270]. Additionally, NO contributes to improved vascularization within bone tissue, further supporting the anabolic effects of osteoblasts. In diabetic conditions, where oxidative stress and inflammation impair osteoblast function, metformin’s ability to activate AMPK and stimulate eNOS expression may help restore normal bone homeostasis [264,271]. In agreement with these findings, in an in vivo study, metformin-treated diabetic rats exhibited increased levels of BMP-2, ALP, and OCN in bone tissue, indicating enhanced osteoblast activity and mineralization. The protective mechanism was attributed to the inhibition of inflammatory signaling through the NF-κB pathway, which often disrupts eNOS function under hyperglycemic conditions [272].

The BMP/Smad signaling pathway is crucial for regulating bone growth and preserving skeletal homeostasis. BMPs, part of the transforming growth factor-beta (TGF-β) family, are critical for osteogenesis, chondrogenesis, and skeletal development. They facilitate DNA synthesis and cellular replication during osteogenic differentiation by activating a receptor complex (known as BMP receptor complex) consisting of type I and type II serine/threonine kinase receptors. Upon BMP interaction, the type II receptor phosphorylates the type I receptor, thereby activating receptor-regulated Smads (R-Smads), specifically Smad1, Smad5, and Smad8. These phosphorylated R-Smads assemble with Smad4, migrate into the nucleus, and modulate the transcription of genes essential for osteoblast development and bone production [273,274]. Metformin has been shown to influence the BMP/Smad signaling axis in bone. In particular, metformin inhibits the phosphorylation of Smad1/5 triggered by BMP6 in osteoblasts, correlating with the overexpression of Smad6. Consistently, this inhibition is attenuated when Smad6 is knocked down, indicating that Smad6 plays a crucial role in this process. Additionally, metformin activates AMPK, which further enhances Smad6 and Smurf1 expression, while reducing ALK2 (activin receptor-like kinase-2, a type I receptor) levels, contributing to the inhibition of BMP signaling [275]. On a separate cell line, metformin has been found to suppress the proliferation and differentiation induced by BMP9 in human fetal lung fibroblast cells through the activation of AMPK and inhibition of ALK1/Smad1/5 signaling. This suggests that metformin’s effects on the BMP/Smad signaling axis can be mediated through AMPK activation and subsequent modulation of Smad proteins [276].

Overall, these last findings indicate that metformin may inhibit the BMP/Smad pathway in certain settings. However, its overall influence on osteogenesis is not straightforward and seems to depend on various factors. While some research points to a suppressive effect of metformin on BMP/Smad signaling, other studies have found that it can actually enhance bone formation, potentially by activating AMPK, engaging the Wnt/β-catenin pathway, or increasing Runx2 activity. The studies generally favor a positive role for metformin in osteogenesis, but there are also reports that do not align with this view. These discrepancies likely arise from differences in experimental design, cell types studied, and the complexity of signaling networks involved. More studies are needed to clarify the specific circumstances under which metformin supports or hinders osteogenesis.

#### 3.5.2. Effects on Osteoclasts

Osteoclasts are essential for bone resorption, as they degrade the bone matrix and release calcium into the bloodstream. Dysregulated osteoclast activity significantly contributes to pathological bone loss, including OP [277]. Emerging evidence suggests that metformin exerts inhibitory effects on osteoclastogenesis and reduces bone resorption through two primary mechanisms.

The first one relies on metformin’s ability to regulate the balance between the receptor activator of nuclear factor κB ligand (RANKL) and osteoprotegerin (OPG; a glycoprotein belonging to the tumor necrosis factor receptor superfamily), two essential factors in osteoclastogenesis [278,279,280]. Generally, RANKL binds to its specific receptor, RANK, on the surface of osteoclasts or their precursors, promoting their differentiation, activation, and survival, while OPG, acting as a “decoy receptor” for RANKL, inhibits osteoclastogenesis. Metformin is thought to disrupt this RANKL-RANK interaction by increasing OPG expression and reducing RANKL levels in osteoblasts. This modulation of the OPG/RANKL ratio is suggested to be a key mechanism through which metformin inhibits osteoclast formation and activity. Furthermore, the altered OPG/RANKL ratio also influences the bone remodeling process and controls the bone mineral density and bone microarchitecture [279,281]. Of note, Mai et al. (2011) [279] demonstrated that osteoblasts treated with metformin exhibited a significantly higher OPG/RANKL ratio, resulting in reduced osteoclast differentiation in vivo and in vitro. Furthermore, in adult male Wistar rats, metformin decreased the experimentally induced periapical bone loss area in the mandibles of the rats by subsequently reducing osteoclast numbers and bone resorption areas, owing to diminished RANKL expression and enhanced OPG expression [282]. In a recent study, the cellular-level effects of metformin in patients with periodontitis were examined. The results indicated that metformin inhibited osteoclast development and downregulated essential genes related to osteoclastogenesis, such as RANKL and macrophage colony-stimulating factor (M-CSF). Moreover, metformin suppressed osteoclastogenesis on both plastic and osseous substrates, in addition to inhibiting bone resorption in monocyte cultures activated by M-CSF and RANKL. This inhibitory effect is probably facilitated by the decrease in RANK expression [283].

The second mechanism involves tartrate-resistant acid phosphatase (TRAP), which is an enzyme predominantly expressed in osteoclasts, with its TRAP 5b isoform being particularly significant as a specific marker for osteoclast number and activity [284,285]. Indeed, TRAP plays a central role in osteoclast-mediated bone resorption, serving as a reliable biochemical indicator for assessing both osteoclast function and the extent of bone degradation. Its enzymatic activity facilitates the breakdown of bone matrix proteins, such as collagen, and contributes to the formation of key structures like the ruffled border and resorption lacuna, which are essential for efficient bone resorption. Furthermore, TRAP generates ROS within osteoclasts, enhancing the enzymatic degradation of matrix components and supporting the overall process of bone remodeling [285,286].

Hyperglycemia has been shown to influence TRAP expression and osteoclast activity, thereby exacerbating bone resorption. In fact, in diabetic conditions, such as those induced experimentally, hyperglycemia enhances TRAP production, which correlates with increased osteoclast function and accelerated degradation of bone tissue [281]. For instance, studies involving streptozotocin (STZ)-induced diabetic rats have reported marked elevations in both TRAP enzymatic activity and mRNA expression, aligning with heightened osteoclast-mediated bone loss [287]. However, findings regarding serum TRAP levels in STZ-injected rats have been inconsistent. Specifically, Guo et al. [287,288] observed a significant reduction in serum TRAP activity in these diabetic models, whereas other researchers reported substantial increases in serum TRAP concentrations [289,290,291]. These discrepancies may reflect differences in experimental protocols, stages of disease progression, or the specific isoforms of TRAP being measured.

Despite these complex and sometimes contradictory findings regarding TRAP levels in diabetic conditions, research suggests potential therapeutic avenues. Within this context, metformin has been shown to exert protective effects on bone health by modulating TRAP levels and suppressing osteoclast activity. Adeyemi et al. demonstrated that oral administration of metformin significantly reduced TRAP levels in a T2DM rat model [292]. Additionally, another study has reported that metformin inhibits osteoclast differentiation and decreases TRAP concentration and activity, further supporting its role in mitigating diabetes-induced bone resorption [293].

#### 3.5.3. Clinical Insights into the Impact of Metformin on Bone Health in Older Adults

One area of concern with metformin use is the risk of lactic acidosis, particularly in the elderly population. However, metformin can be used safely in elderly patients with osteoporosis if renal function is carefully monitored. The American Diabetes Association (ADA) recommends metformin for older adults with an estimated glomerular filtration rate (eGFR) ≥ 30 mL/min/1.73 m^2^, with dose adjustments for those with an eGFR between 30–45 mL/min/1.73 m^2^ [294]. Recent large cohort studies and systematic reviews have further clarified the safety profile of metformin in elderly patients with osteoporosis and renal dysfunction. These studies indicate that metformin is not associated with an increased risk of lactic acidosis in patients with stable chronic kidney disease (CKD) and an eGFR ≥ 30 mL/min/1.73 m^2^. However, the risk of lactic acidosis rises significantly when eGFR falls below this threshold. Multiple large observational studies and meta-analyses have demonstrated that the incidence of lactic acidosis in metformin users with an eGFR ≥ 30 mL/min/1.73 m^2^ is comparable to the background rate in the general diabetic population and is not higher than in individuals not taking metformin. In summary, metformin is generally safe for elderly patients with osteoporosis, provided renal function is stable and eGFR is ≥30 mL/min/1.73 m^2^. However, it should be avoided or discontinued in patients with more advanced renal dysfunction due to the increased risk of lactic acidosis [295,296,297].

There has been limited clinical study on the impact of metformin on bone mineral density (BMD) and/or fracture risk in the elderly. One such investigation assessed the influence of different metformin dosages on BMD and bone metabolism in older male patients with T2DM. Participants were treated with either a low dose (0.5 g twice daily) or a high dose (0.5 g four times daily) of metformin for 12 weeks. BMD measurements focused on the lumbar vertebrae (L1–L4) and hip regions, alongside assessments of bone metabolic markers. Post-treatment results indicated significant improvements in BMD and bone metabolic parameters in the high-dose metformin group compared to the control group. Specifically, in the high-dose metformin group, BMD and 25-hydroxyvitamin D levels were significantly elevated, while markers of bone resorption, such as N-terminal midfragment and β-isomerized C-terminal telopeptides, decreased substantially.

Collectively, these findings suggest that higher doses of metformin not only improve glycemic control but also promote bone health by increasing BMD and favorably altering bone metabolism. This underscores its potential therapeutic significance in managing osteoporosis in elderly males with T2DM [298].

In a distinct yet similar clinical trial, researchers investigated the effectiveness of combining dapagliflozin with metformin in older individuals with T2DM and OP. Patients were divided into two groups: metformin alone (1 g/day) and dapagliflozin + metformin, with treatment lasting 12 months. Both groups showed improvements in BMD at L1–L4, femoral neck (FN), and total hip (TH), as well as in bone metabolic indicators and visual analog scale (VAS) scores. Nevertheless, the combination therapy group had markedly greater improvements in bone mineral density, a reduced fracture incidence, and superior overall efficacy [299].

Finally, an extensive cohort study in China involving patients with T2DM (mean age 60 years) revealed that metformin treatment was linked to enhanced bone health outcomes. Specifically, metformin intake was associated with elevated T-scores at the FN and TH, along with decreased odds ratios for osteopenia and OP. The results showed the greatest difference in the females, underscoring metformin’s promise in alleviating low BMD and OP risk in individuals with T2DM [257].

To conclude, it appears that metformin’s effects on bone health may not be solely attributable to improved glycemic control in diabetes models. Evidence suggests that metformin may also improve bone health independently of its effects on diabetes, although this distinction remains an area of ongoing investigation.

Clinical studies provide some evidence of a causal relationship between metformin use and reduced osteoporosis risk, increased BMD, and lower fracture incidence, independent of diabetes status or glycemic control. These studies, which use genetic proxies for metformin exposure, help minimize confounding by diabetes or metabolic status, supporting the possibility of a direct effect on bone health. However, further studies are needed to confirm these findings [258,300]. Additionally, two meta-analyses, including studies involving non-diabetic populations, indicate that metformin reduces bone turnover markers. While its effects on BMD are modest and may not reach statistical significance in all studies, the observed bone-protective actions in both diabetic and non-diabetic contexts suggest a potential direct influence on bone metabolism [301,302].

As discussed earlier, mechanistic in vitro and in vivo studies in non-diabetic models provide further support for this hypothesis. Metformin has been shown to directly modulate bone cell function by activating AMPK in osteoblasts and osteocytes, upregulating anabolic pathways such as Wnt signaling and Runx2, and suppressing osteoclastogenesis through reduced RANKL expression. Notably, these effects are observed in the absence of hyperglycemia and in non-diabetic animals and cell cultures, lending weight to the argument for a direct mechanism [303,304,305].

In summary, while metformin’s bone-protective effects are investigated in both diabetic and non-diabetic contexts, it remains uncertain to what extent these effects are independent of its glycemic control properties. Further research is needed to clarify whether metformin’s benefits on bone health are primarily direct or mediated through its impact on diabetes-related bone loss [258,300,303,304,305].

## 4. Conclusions

**Figure 5:**
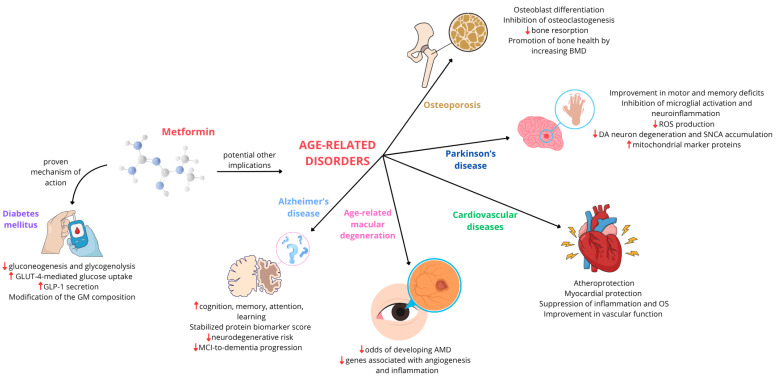
The potential anti-aging effects of metformin. Beyond its established antihyperglycemic action in diabetes mellitus —achieved by reducing hepatic gluconeogenesis and glycogenolysis, increasing GLUT-4-mediated glucose uptake, enhancing GLP-1 secretion, and modifying the GM composition—evidence suggests a potential role for metformin in age-related diseases. In Alzheimer’s disease, metformin improves cognition, memory, attention, and learning, stabilizes protein biomarker scores, and reduces neurodegenerative risk and progression from MCI-to-dementia. In age-related macular degeneration, this drug reduces the risk of developing AMD and suppresses the expression of genes associated with angiogenesis and inflammation. In cardiovascular diseases, metformin favours atheroprotection and myocardial protection, suppresses inflammation and OS, and improves vascular function. In Parkinson’s disease, it improves motor and memory deficits; inhibits microglial activation and neuroinflammation; reduces ROS production, DA neuron degeneration, and SNCA accumulation; and increases the expression of mitochondrial marker proteins. Finally, in osteoporosis, metformin favours osteoblast differentiation, inhibits osteoclastogenesis, reduces bone resorption, and promotes bone health by increasing BMD.

This review summarizes the anti-aging effects of the oral antihyperglycemic drug metformin (Figure 5). Overall, metformin appears to be safe, well-tolerated, and commercially available, with important limitations. Compelling evidence links metformin intake to reduced neurodegenerative symptoms, regulation of AMD progression, cardiovascular benefits, increased bone mineral density, and reduced risk of bone fractures. However, contradictory data exist and must be considered. Factors such as ethnicity, diet, lifestyle, and sex identity have emerged as confounding variables in many clinical trials reviewed here. In this context, metformin’s performance in diseases with a particular sex-biased incidence, such as osteoporosis or dementia, might be biased by gender-specific effects. Crucially, participants’ age, comorbidities, and clinical history also significantly impact metformin’s pharmacodynamics and pharmacokinetics. In this context, synergism or antagonism with concomitantly prescribed drugs, a common situation in old people, remains under investigated. To address these issues, further studies should include both young and aged individuals. This would allow one to (1) address metformin’s potential for lifelong prevention treatment; (2) determine the optimal age for treatment initiation; and (3) assess the impact of concomitant drug prescription on metformin efficacy. At the same time, a consensus on intervention timing, dose, and treatment formulations should be reached within the scientific community to limit inter-study variability. In sum, our review clearly outlines metformin’s potential to mediate beneficial effects against major age-related diseases, yet inconclusive or even negative studies must also be considered. Ultimately, a deeper understanding of metformin’s context-dependent mechanisms, combined with tailored clinical approaches, will be crucial to maximize its therapeutic impact across diverse aging-related disorders.

## Figures and Tables

**Table 1:** Metformin therapy in AD: overview from clinical studies.

Database/Centre/Study	Cohort	Main Outcome	Reference
VALCODIS Cohort	N = 213 T2DM (50–80 y.o.)	↓ p-tau (CSF)	[62]
National Alzheimer’s Coordinating Center (NACC)	N = 48,605 MCI (>39 y.o.)	↓ MCI-to-dementia progression	[63]
NCT01965756	N = 48,605 MCI	Stabilized protein biomarker score (AZU1, CASP-3, CCL11, CCL20, IL32, PRTN3, and REG1A) (plasma + CSF)	[64]
N.A.	N = 527,138 (middle-aged)	↓ mitochondrial complex I (brain cortex)	[65]
ADNI study	N = 6937 MCI-AD (55–90 y.o.)	↑ Cognition, cortical thickness, hippocampal volume = glycemia, triglycerides, cholesterol	[66]
Korean National Health Insurance Service DM cohort	N= 10,050 T2DM (n = 1675 AD) (50–85 y.o.)	↑ Cognition	[67]
National Alzheimer’s Coordinating Center database	N = 1999 T2DM (n = 807 AD)	↑ Memory (in T2DM non-AD)	[68]
FAERS database	N = 66,085 (>64 y.o.) T2DM (n = 1250 AD)	No improvement	[69]
Korean cohort registry	N = 732 (>59 y.o.)	↓ MMSE, verbal scores = daily activity index	[70]
US Veterans Affairs electronic medical record	N = 5528 T2DM (>49 y.o.)	↓ Neurodegenerative risk	[71]
University of Pennsylvania Health System (UPHS)	N = 20 MCI (55–80 y.o.)	↑ Execution performance, memory, attention, learning ↑/= orbitofrontal cerebral blood flow	[72]
Columbia University Medical Center	N = 80 MCI (55–90 y.o.) overweight/obese	↑ Selective Reminding Test (SRT)	[73]
United Kingdom-based General Practice Research Database (GPRD)	N = 7086 AD (>65 y.o.)	↑ AD risk	[74]
Swedish Registry for Cognitive/Dementia Disorders (SveDem)	N = 15,428 AD (77.29 mean y.o.)	↑ Cognition	[75]
National Alzheimer’s Coordinating Center database	N = 1393 T2DM (>49 y.o.)	↓ AD risk	[76]
ICES Database, Ontario, Canada	N = 34,700 T2DM (>65 y.o.)	= AD risk	[77]

Abbreviations: AD: Alzheimer’s disease; AZU1: azurocidin 1; CASP-3: caspase 3; CCL11: C-C motif chemokine 11; CCL20: C-C motif chemokine ligand 20; CSF: cerebrospinal fluid; IL32: interleukin 32; MCI: mild cognitive impairment; MMSE: mini-mental state examination; n: number of participants within N; N: total number of participants; N.A: not applicable; PRTN3: proteinase 3; REG1A: regenerating family member 1 alpha; T2DM: type 2 diabetes mellitus; y.o.: years old; ↑ increase; ↓ decrease; = no change.

**Table 3:** Preclinical and clinical studies of metformin therapy in cardiovascular disorders.

Preclinical Studies			
Mechanism	Model	Main Outcome	Reference
Activation of AMPK/autophagy axis (atheroprotection)	ApoE(−/−) mice	Attenuation of Ang-II-induced atheromatous plaque formation and aortic aneurysm partly	[187]
	Normoglycemic *Ldlr*−/− hyperlipidaemic mice	Suppression of atherogenesis	[188]
	GFP-LC3 transgenic mice by PCSK9	Improvement in autophagy-mediated cholesterol efflux	[189]
Suppression of mitochondrial ROS-mediated pro-inflammatory pathway (atheroprotection)	Male Sprague–Dawley rats treated with glucose/metformin	Alteration in cellular redox state and reduction in glucose production by inhibiting complex IV	[190]
	Mice exposed to PM and treated with metformin	Prevention in PM-induced generation of METC-ROS	[191]
Activation of sirtuin signaling (myocardial protection)	Male C57BL/6J *Sirt2* knockout mice	Activation of AMPK signaling Protection against cardiac hypertrophy	[192]
	2-Hit PH-HFpEF model in rats with multiple features of metabolic syndrome due to a double-leptin receptor defect (obese ZSF1)	Normalization of pulmonary hypertension associated with HF	[193]
	Salt-induced hepatic inflammation in mouse model	Amelioration of high-salt diet-induced hepatic inflammation and myocardial damage	[194]
Activation of autophagy-dependent pathway (myocardial protection)	Diabetic mice	Prevention of cardiomyopathy	[195]
	Carfilzomib-induced cardiotoxicity in mice	Protection against carfilzomib-induced cardiotoxicity	[196]
	Fly stocks	Activation of autophagy	[197]
**Clinical Studies**			
**Database/Centre** **Cohort**	**Metformin Treatment**	**Main Outcome**	**Reference**
n = 33 non-diabetic women with angina	n = 16 patients: 0.5 g twice daily for 8 weeks	Improvement in vascular function Decrease in myocardial ischemia	[198]
CAMERA study: n = 173 non-diabetic patients with coronary heart disease and on statin therapy	n = 86 patients: 850 mg twice daily for 18 months	No significant effect on cIMT Reduction in body weight, body fat, BMI, and waist circumference	[199]
DPPOS study: n = 3234 individuals with pre-diabetes	n = 926 patients: 850 mg twice daily (14 years of follow-up)	Reduction in CAC only in male subjects	[200]
CODYCE study: n = 258 propensity-matched patients with stable angina	n = 86 patients: 850 mg twice a day (6–12–24 months of follow-up)	Suppression of inflammation and oxidative stress	[201]
n = 849 non-diabetic patients with an inflammatory disease treated with continuous prednisolone	n = 26 patients: 850 mg/day for the first 5 days, 850 mg twice a day for the next 5 days, and 850 mg three times a day subsequently for 12 weeks	Reduction of metabolic complications, inflammation, LDL-cholesterol, and cIMT	[202]
REMOVAL study: n = 428 patients with T1DM (40 years and older)	n = 219 patients: 1 g twice daily for 3 years	Reduction in the maximal cIMT, body weight and blood LDL-cholesterol No adequate improvement in glycemic control	[203]
EMERALD study: n = 48 T1DM adolescents (12–21 years)	n = 25 patients: 2000 mg daily for 3 months	Reduction in cIMT, BMI, and fat mass Improvement in aortic dysfunction	[204]
n = 380 patients with diabetes and HF	n = 87 patients: 1000 mg (29 patients), lower than 1000 mg (18 patients), between 1000 and 2000 mg (14 patients), and 2000 mg (25 patients) daily or higher	Signs of more stable HF (lower BNP levels, reduced mitral and tricuspid regurgitation severity, improved left and right ventricular function, and decreased diuretic dosage) Increase in survival	[205]
SAVOR-TIMI 53 trial: n = 12,156 patients with T2DM and high cardiovascular risk with or without HF or kidney dysfunction	n = 8971 patients exposed to metformin	Lower risk of all-cause mortality and cardiovascular death No difference in risk of the composite endpoint of cardiovascular death, myocardial infarction, or ischemic stroke	[206]
n = 36 patients with HF	n = 19 patients: 1450 ± 550 mg/day for 3 months	Increase in relative efficiency Reduction in myocardial oxygen consumption Preservation of cardiac stroke work No effects on resting and exercise ejection fraction, global longitudinal strain, and exercise capacity	[207]
MET-REMODEL study: N = 68 patients without diabetes, but with CAD, LVH, IR, and/or pre-diabetes	n = 34 patients: 2000 mg daily for 12 months	Reduction in LVMI, LVM, body weight, subcutaneous adipose tissue, office systolic blood pressure, and in concentration of thiobarbituric acid reactive substances	[208]
MET-DIME trial: N = 54 non-diabetic patients with diastolic dysfunction	n = 27 patients: 1000 mg twice daily (2 years of follow-up)	Improvement in e-wave’ velocity Reduction in the HOMA-IR index No effect on LVMI, and hs-CRP and N-terminal -pro-BNP plasma levels	[209]

Abbreviations: AMPK: AMP-activated protein kinase; AngII: angiotensin II; ApoE: apolipoprotein E; BNP: brain natriuretic peptide; BMI: body-mass index; CAC: coronary artery calcium; CAD: coronary artery disease; cIMT: carotid intima-media thickness; GFP-LC3: microtubule-associated proteins 1A/1B light chain 3 tagged with green fluorescent protein (GFP); HF: heart failure; HOMA-IR: homeostasis model assessment of insulin resistance; hs-CRP: high sensitivity C-reactive protein; IR: insulin resistance; LDL: low-density lipoprotein; Ldlr: low-density lipoprotein receptor; LVH: left ventricular hypertrophy; LVM: left ventricular mass; LVMI: left ventricular mass indexed; METC: mitochondrial electron transport chain; PCSK9: proprotein convertase subtilisin/kexin type 9; PH-HFpEF: pulmonary hypertension-heart failure with preserved ejection fraction; PM: particulate matter; ROS: reactive oxygen species; Sirt2: sirtuin 2; T1DM: type 1 diabetes; T2DM: type 2 diabetes mellitus.

**Table 4:** Summary of studies investigating the association between metformin exposure and AMD development in individuals with DM.

Type of Study, Follow-Up Periods (yrs), Patient Characteristics (Age Mean in Yrs (SD) N. Participants (Exposed/Nonexposed))	Daily Metformin Dosage (mg)	AMD Type	Main Outcome	Reference
Case-control in the United States, 2 yrs, 74.9 yrs (10.3), 81,262/79,497	Various doses, mean: 163	N.A.	Metformin use was associated with reduced odds of developing AMD. This association was dose dependent, with low to moderate doses of metformin showing the greatest potential benefit.	[232]
Case-control in the United States, N.A, N.A, Control: 77 Case: 75.09	N.A.	Dry and wet	Patients who had taken metformin had decreased odds of developing AMD, suggesting that metformin may have a therapeutic role in AMD development or progression in those who are at risk.	[233]
Retrospective cohort in Taiwan, 6.7 yrs, 56.1 yrs (12.6), 45,524/22,681	Various doses (400–2100)	N.A.	Metformin use was associated with reduced odds of developing AMD. Also, the trend of a significantly lower AMD risk was found with a higher dose of metformin.	[234]
Retrospective cohort in United States, N.A, 67.5 yrs (8.9), 166,115/841,111	Various doses, mean 1000	Dry	There is not sufficient evidence to suggest that metformin has a meaningful impact on the development of AMD	[235]
Retrospective cohort in United Kingdom, 5.7 yrs (4.1), 62.8 yrs (11.6), 154,016/19,673	N.A.	Dry and Wet	There is not sufficient evidence to suggest that metformin has a meaningful impact on the development of AMD	[236]
Retrospective cohort in Taiwan, 5 yrs, 62.06 yrs old (8.83), 377,873/350,825	Various doses	N.A.	Metformin use is associated with a dose-dependent risk of AMD in patients with DM. Indeed, the greater benefit is observed at lowest dose, while the highest dose exhibited an increased risk of AMD.	[237]
Retrospective cohort in China, 6 months to 10 yrs, Median 67 yrs, 209/115	More than 250	Any type	Among patients with DM for ≥10 years, metformin users were less likely to develop any AMD and early AMD than non-users	[238]
Retrospective Cohort in Taiwan, 5.6 yrs, 65.04 yrs (8.3), 13,303/13,303	Various doses	N.A.	The results of this study supported a lower risk of AMD in ever users of metformin when compared to never users	[239]

Abbreviations: AMD: age-related macular degeneration, DM: diabetes mellitus, mg: milligram, N.A.: not applicable, SD: standard deviation, yrs: years.

## Data Availability

Not applicable.

## Abbreviations

AMD: age-related macular degeneration; BMD: bone mineral density; DA: dopamine; GLP-1: glucagon-like peptide-1; GLUT-4: glucose transporter 4; GM: gut microbiota; MCI: mild cognitive impairment; OS: oxidative stress; ROS: reactive oxygen species; SNCA: alpha synuclein (Created with Canva).
